# WAC Facilitates Mitophagy‐mediated MSC Osteogenesis and New Bone Formation via Protecting PINK1 from Ubiquitination‐Dependent Degradation

**DOI:** 10.1002/advs.202404107

**Published:** 2024-11-18

**Authors:** Shuai Fan, Jinteng Li, Guan Zheng, Ziyue Ma, Xiaoshuai Peng, Zhongyu Xie, Wenjie Liu, Wenhui Yu, Jiajie Lin, Zepeng Su, Peitao Xu, Peng Wang, Yanfeng Wu, Huiyong Shen, Guiwen Ye

**Affiliations:** ^1^ Department of Orthopedics The Eighth Affiliated Hospital Sun Yat‐sen University Shenzhen 518033 P. R. China; ^2^ Center for Biotherapy The Eighth Affiliated Hospital Sun Yat‐sen University Shenzhen 518033 P. R. China

**Keywords:** mesenchymal stem cell, mitophagy, osteogenesis, PINK1, WAC

## Abstract

Osteogenic differentiation of mesenchymal stem cells (MSCs) plays a pivotal role in the pathogenesis and treatment of bone‐related conditions such as osteoporosis and bone regeneration. While the WW domain‐containing coiled‐coil adaptor (WAC) protein is primarily associated with transcriptional regulation and autophagy, its involvement in MSC osteogenesis remains unclear. Here, the data reveal that the levels of WAC are diminished in both osteoporosis patients and osteoporosis mouse models. It plays a pivotal function in facilitating MSC osteogenesis and enhancing new bone formation both in vitro and in vivo. Mechanistically, WAC promotes MSC osteogenesis by protecting PINK1, a crucial initiator of mitophagy, from ubiquitination‐dependent degradation thereby activating mitophagy. Interestingly, WAC interacts with the TM domains of PINK1 and prevents the K137 site from ubiquitination modification. The study elucidates the mechanism by which WAC modulates MSC osteogenesis, binds to PINK1 to protect it from ubiquitination, and identifies potential therapeutic targets for osteoporosis and bone defect repair.

## Graphical abstract

WAC binds to PINK1, shielding it from ubiquitination degradation, thereby maintaining normal levels of mitophagy and positively regulating the osteogenic differentiation of MSCs. The absence of WAC results in the ubiquitination degradation of PINK1 at the K137 locus and a reduction in mitophagy levels, leading to impaired MSC osteogenesis.

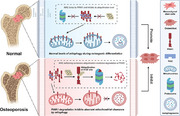

## 1 Introduction

Mesenchymal stem cells (MSCs) possess pluripotent capabilities, differentiating into osteoblasts, chondrocytes, and adipocytes.^[^ ^1^, ^2^ ^]^ In the context of bone homeostasis, MSCs contribute to bone development as osteoblasts or influence bone metabolism through adipocyte differentiation.^[^ ^3^, ^4^, ^5^ ^]^ The differentiation of MSCs is integral to maintaining bone homeostasis, positioning MSCs as a significant tool in bone regeneration and tissue engineering techniques.^[^ ^6^, ^7^, ^8^, ^9^ ^]^ In orthopedic conditions like osteoporosis, MSCs can stimulate bone formation and aid in the repair of damaged bone tissue by undergoing osteoblastic differentiation.^[^ ^10^, ^11^, ^12^ ^]^ However, the molecular mechanisms governing the osteogenic differentiation of MSCs remain largely elusive, posing a challenge to the advancement of MSCs‐based cell therapies for clinical applications in osteoporosis, bone defects, and related diseases.

The WW domain‐containing adaptor with coiled‐coil (WAC) protein is localized in the nucleus and Golgi, and is characterized by protein‐protein interactions through the N‐terminal WW region and the C‐terminal convoluted helix region.^[^ ^13^, ^14^ ^]^ Specifically, WAC acts as a binding chaperone for RNF20/40, assisting in the regulation of H2B ubiquitination and, consequently, transcription.^[^ ^15^ ^]^ WAC has also been identified as a potent positive regulator of autophagy. Nicole et al. observed a significant reduction in LC3 lipidation levels and an increase in protein levels of P62 upon WAC knockdown in 293T cells.^[^ ^16^ ^]^ Justin et al. found that WAC regulates autophagosome formation by binding to GM130.^[^ ^13^ ^]^ Existing studies primarily suggest that WAC is associated with central nervous system disorders, such as epilepsy and mental retardation.^[^ ^17^ ^]^ However, there is a lack of knowledge about WAC in bone metabolism‐related diseases, especially osteoporosis.

Mitochondria, crucial for biological oxidation and energy conversion, participate in essential cellular processes like intracellular homeostasis, proliferation, senescence, and apoptosis.^[^ ^18^ ^]^ Susceptible to damage due to the absence of histone protection and limited self‐repair mechanisms, mitochondria respond to stimuli like hypoxia and free radicals. In cases where damage is irreparable, mitochondria initiate mitophagy for self‐degradation, eliminating excess dysfunctional mitochondria to sustain cellular homeostasis and normal functions.^[^ ^19^ ^]^ Dysfunctional mitophagy is linked to degenerative diseases such as Parkinson's,^[^ ^20^, ^21^ ^]^ Alzheimer's,^[^ ^22^ ^]^ and cancer.^[^ ^23^, ^24^ ^]^ Recent studies reveal that dysregulation of autophagy and mitophagy disrupts bone metabolism homeostasis,^[^ ^25^ ^]^ significantly impacting osteogenesis and reducing the expression of bone formation proteins like BMP2 and collagen I in MSCs.^[^ ^26^ ^]^

PINK1, belonging to the serine/threonine kinase family, is situated on the outer mitochondrial membrane, playing a crucial role in monitoring mitochondrial status.^[^ ^27^ ^]^ Numerous studies have illustrated that PINK1 initiates mitochondrial autophagy, with the PINK1‐Parkin‐mediated pathway considered the most pivotal in this process.^[^ ^28^ ^]^ Studies have demonstrated that PINK1 deficiency disrupts mitochondrial homeostasis and hinders osteoblast differentiation.^[^ ^29^ ^]^ However, the mechanism of regulation of mitophagy in osteogenesis is unclear.

In this study, our objective is to explore the function and mechanism of WAC in regulating the osteogenesis ability of MSCs. PINK1 is situated downstream of WAC, and WAC enhances the MSC osteogenesis by impeding the ubiquitinated degradation of PINK1 and fostering the level of mitophagy. Our findings propose that WAC positively modulates the osteogenic process of MSCs, both in vivo and in vitro, making it a promising target for osteoporosis and bone defect repair.

## 2 Results

### 2.1 The Level of WAC Is Upregulated During MSC Osteogenesis and Decreased in Osteoporosis

**Figure 1:**
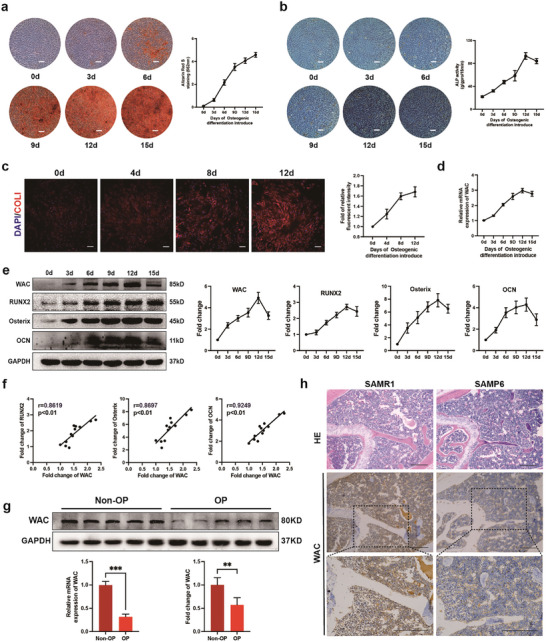
WAC was up‐regulated during the osteogenic differentiation of MSCs. a) ARS Staining and quantification throughout MSC osteogenic differentiation; b) ALP Staining and quantification during the progression of MSC osteogenesis; c) Immunofluorescence Staining for COL1 (red) during osteogenic induction. Quantification is presented in the right panel (Scale bar  =  50 µm); d) Relative mRNA levels of WAC assessed by qRT‐PCR at different time points during MSC osteogenesis; e) Protein levels of WAC and osteogenic markers (RUNX2, Osterix, OCN) during MSC osteogenic differentiation; f) Pearson correlation analysis depicting the relationship between WAC expression and quantification of RUNX2, Osterix, and OCN levels during MSC osteogenic differentiation; g) qRT–PCR and Western blotting to detect WAC mRNA and protein levels in bone marrow MSCs from nonosteoporotic patients and patients with osteoporosis; h) HE staining and immunohistochemical staining for WAC in the femurs of SAMR1 mice and SAMP6 mice (Scale bar  =  100 µm). All data are presented as the means ± SD, *n* = 6 per group in (a, b, c), *n* = 5 in (g), *n* = 9 in (d, e, g), *n* = 12 in (f). Statistical differences were determined using Student's *t*‐test or ANOVA. ^**^ *p* < 0.01 and ^***^ *p* < 0.001.

We first cultured MSCs in osteogenic differentiation medium (OM) and determined the osteogenic differentiation potential at different time points. ARS staining showed a gradual increase in calcium nodules from day 0 to day 15, and ALP staining peaked on day 12 and then decreased (**Figure** **1**a,b). Immunofluorescence assay showed that the red fluorescence intensity of COL1 gradually increased with osteogenesis of MSCs (Figure 1c). These results indicated successful induction of osteogenic differentiation. Subsequently, we examined the level of WAC during the MSC osteogenesis. Both the mRNA and protein levels of WAC increased with days of osteogenesis (Figure 1d,e). Correlation analysis showed that the level of WAC was positively correlated with the expression of osteogenic markers including RUNX2, Osterix and OCN (Figure 1f). In addition, we collected MSCs from osteoporosis patients (OP) and found that both the mRNA and protein levels of WAC were down‐regulated in OP compared to non‐OP samples (Figure 1g). Senescence‐Accelerated Mouse Prone 6 (SAMP6) mice exhibit significant and progressive bone loss due to impaired osteoblast formation, characterized by low trabecular bone formation and reduced bone density, starting at 4 months of age, which makes SAMP6 an ideal model for studying senile osteoporosis.^[^ ^30^ ^]^ We further examined the differences in WAC levels in bone tissue between SAMP6 mice and Senescence‐Accelerated Mouse Resistant 1 (SAMR1) control mice. Immunohistochemistry revealed a significant reduction in WAC levels in the bone tissue of SAMP6 mice compared to SAMR1 mice (Figure 1h). Together, these data suggest an important role of WAC in MSC osteogenesis and osteoporosis.

### 2.2 WAC Positively Regulates Osteogenic Differentiation of MSCs In Vitro

**Figure 2:**
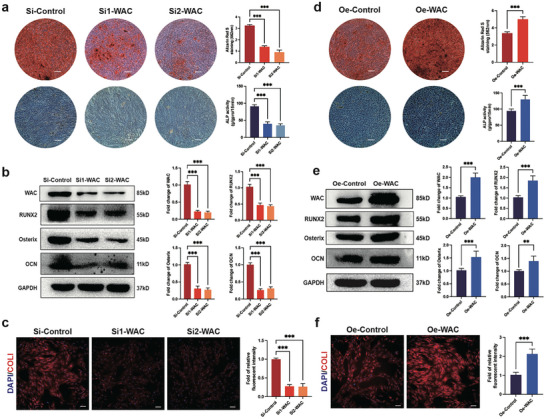
WAC positively regulated the osteogenic differentiation of MSCs in vitro. WAC is modulated in MSCs through SiRNA and Lentivirus. a) MSCs were cultured in osteogenic medium after transfection with SiRNA. ARS staining, ALP staining, and quantification were performed on day 12; b) Western blotting for protein levels of osteogenesis‐related markers (RUNX2, Osterix, OCN). Quantification is presented in the right panel; c) Immunofluorescence staining for COL1 (red) after SiRNA transfection. Quantification is shown in the right panel (Scale bar  =  50 µm); d) Overexpression lentivirus of WAC transfected into MSCs, followed by culture in osteogenic medium. ARS staining, ALP staining, and quantification conducted on day 12; e) Western blotting for protein levels of osteogenesis‐related markers after overexpression lentivirus transfection; f) Immunofluorescence staining for COL1 (red) after overexpression lentivirus transfection. Quantification is shown in the right panel (Scale bar  =  50 µm). All data are presented as the means ± SD, *n* = 6 per group in (a, c, d, f), *n* = 9 per group in (b, e). Statistical differences were determined using Student's *t*‐test or ANOVA. ns not statistically significant, ^**^ *p* < 0.01 and ^***^ *p* < 0.001.

To assess the impact of WAC on the osteogenic differentiation of MSCs, we employed SiRNA and overexpressed lentivirus to respectively down‐regulate and up‐regulate WAC expression in MSCs. Subsequently, we evaluated the osteogenic capacity of MSCs among different groups following 12 days of osteogenic induction. ARS staining indicated that WAC knockdown led to a decrease in the formation of calcium nodules, with ALP staining showing reduced and less intense staining (**Figure** **2**a). Additionally, the knockdown of WAC significantly lowered the protein levels of osteogenesis markers (RUNX2, Osterix, OCN) (Figure 2b). Immunofluorescence analysis demonstrated a decrease in the red fluorescence intensity of COL1 after WAC knockdown, signifying impaired osteogenesis (Figure 2c). Conversely, overexpression of WAC resulted in an opposite effect, with increased calcium nodule formation observed during osteogenesis in ARS staining. ALP assay results were in line with ARS staining, revealing denser and deeper staining with WAC overexpression during osteogenesis (Figure 2d). Elevated protein levels of osteogenic differentiation markers were also evident upon WAC overexpression (Figure 2e). Immunofluorescence analysis similarly showed enhanced fluorescence intensity of COL1 after WAC overexpression (Figure 2f). In conclusion, these findings strongly suggest that WAC in MSCs has the potential to promote the osteogenic differentiation of MSCs in vitro.

### 2.3 WAC Regulates Osteogenic Differentiation of MSCs Through Mitochondrial Autophagy

WAC primarily participates in uH2B modification and autophagy regulation.^[^ ^15^, ^16^ ^]^ In our initial findings, we observed no significant alteration in the uH2B modification levels on the DNA of several crucial osteogenic factors following WAC knockdown, as determined by CUT&TAG assay (Figure S1, Supporting Information). This implies that WAC may not play a role in regulating MSC osteogenesis through uH2B modification of key osteogenic factors. In light of existing studies suggesting the involvement of WAC in autophagy and mitophagy processes, we hypothesized that WAC might regulate the osteogenic differentiation of MSCs through the mitochondrial autophagy‐related pathway. Initially, we assessed the protein levels of mitophagy markers (PINK1, P62, VDAC, TIMM23, LC3B) at different time points during MSC osteogenesis. The results illustrated a concurrent increase in mitophagy levels as MSCs underwent osteogenic differentiation (Figure S2a, Supporting Information).

**Figure 3:**
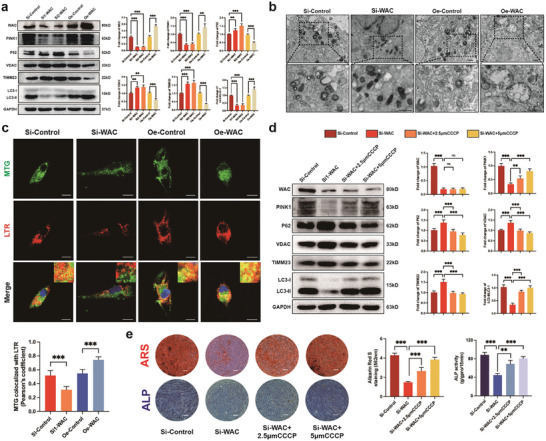
WAC regulates osteogenic differentiation of MSCs through mitophagy. a) Protein levels of mitochondrial autophagy‐associated markers (PINK1, P62, VDAC, TIMM23, and LC3B) were assessed through Western blotting after WAC knockdown or overexpression. Quantification of the data is depicted in the right panel; b) Utilizing transmission electron microscopy (TEM) to reveal mitochondrion and autophagosomes in MSCs under osteogenic differentiation‐inducing conditions. (Scale bar  =  1 µm); c) Confocal microscopy‐based co‐localization analysis of MitoTracker Green (MTG) and LysoTracker Red (LTR), with cell nuclei stained using Hoechst 33 342 (blue) and the Pearson's coefficient was calculated from the image using ImageJ software and the data are shown in the panel below (Scale bar  =  20 µm); d) Following knockdown of WAC and treatment with various CCCP, detection of protein levels of mitochondrial autophagy‐related markers; e) WAC was subjected to knockdown followed by treatment with varying concentrations of CCCP. Subsequent staining with ARS and ALP was performed, and data quantification is illustrated on the right. Quantification of the data is presented in the right panel. All data are presented as the means ± SD, *n* = 9 per group. Statistical differences were determined using Student's *t*‐test or ANOVA. ns not statistically significant, ^**^ *p* < 0.01 and ^***^ *p* < 0.001.

Subsequently, the analysis of protein levels of mitophagy markers revealed a decrease in mitophagy during MSC osteogenesis with WAC knockdown and an increase with WAC overexpression (**Figure** **3**a). Additionally, TEM unveiled changes in mitochondrial morphology and the accumulation of autophagosomes during osteogenesis. With the overexpression of WAC, the abundance of autophagic vesicles increased, and well‐defined mitochondria were scarcely visible. Conversely, WAC knockdown MSCs displayed only a limited number of distorted mitochondria and autophagosomes (Figure 3b). During the osteogenic differentiation of MSCs, a certain level of co‐localization of MTG (MitoTracker Green) and LTR (LysoTracker Red) was observed. This co‐localization decreased with WAC knockdown and increased with WAC overexpression (Figure 3c). Immunofluorescence analysis further revealed that the green fluorescence of LC3B diminished with WAC knockdown and enhanced with WAC overexpression (Figure S3, Supporting Information). In summary, the level of mitophagy increased during MSC osteogenesis, and WAC deficiency decreased this level, while overexpression of WAC promoted mitophagy.

To verify whether WAC promotes MSC osteogenesis through mitophagy, we employed the mitophagy‐inducing drug Carbonyl cyanide 3‐chlorophenylhydrazone (CCCP). CCCP alleviated the impairment of mitophagy levels caused by SiWAC compared to the control group (Figure 3d). In addition, the osteogenic differentiation level of MSCs was restored to varying degrees with an elevated concentration of CCCP, as evidenced by ARS and ALP staining (Figure 3e). Correspondingly, the protein levels of osteogenic markers also increased (Figure S2b, Supporting Information). Additionally, we observed that the application of the mitophagy inhibitor Mdivi‐1 reversed the impact of WAC overexpression on the MSC osteogenesis (Figure S2c–e, Supporting Information). Together, these data illustrate that WAC regulates MSC osteogenesis through the mitophagy pathway. We further examined the effects of WAC on mitochondrial dynamics and mitochondrial function. Western blotting showed that knockdown of WAC increased the levels of MFN1 (Mitofusin‐1), and increased the levels of DRP1 (dynamin‐related protein 1) and FIS1 (Mitochondrial Fission 1) protein levels (Figure S4a, Supporting Information). These results suggest that WAC promotes mitochondrial fission and inhibits mitochondrial fusion during osteogenic differentiation of MSCs. In addition, after WAC knockdown, ATP levels in MSCs and mitochondrial membrane potential decreased, and accumulated ROS levels increased. In contrast, after WAC overexpression, ATP level and mitochondrial membrane potential increased, and ROS levels decreased (Figure S4b–d, Supporting Information). Overall, these results suggest that WAC is able to maintain normal mitochondrial function during osteogenic differentiation of MSCs.

### 2.4 WAC Regulates Mitophagy via PINK1 to Modulate MSC Osteogenesis

**Figure 4:**
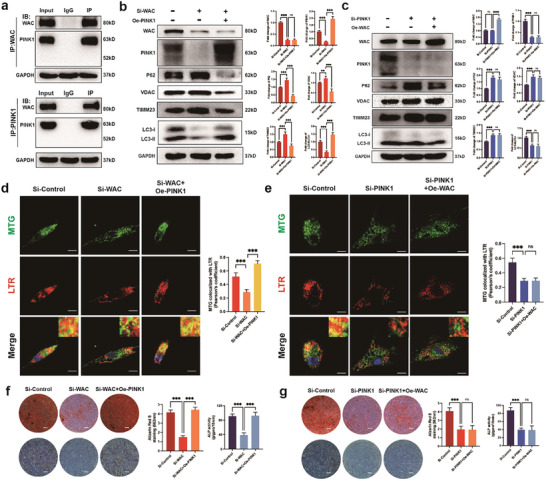
WAC regulates mitophagy via PINK1 to modulate MSC osteogenesis. a) Immunoprecipitation assays using WAC, PINK1, or IgG antibodies on MSCs cell lysates to reveal endogenous interactions of WAC and PINK1. The interactions were detected by Western blotting; b) Detection of mitochondrial autophagy‐related markers after the knockdown of WAC and overexpression of PINK1 and data quantification are presented in the right panel; c) Mitochondrial autophagy‐related markers were detected after the knockdown of PINK1 and overexpression of WAC and data quantification are shown in the right panel; d) Co‐localization analysis of MitoTracker Green and LysoTracker Red, staining of nuclei using Hoechst 33 342 (blue) after WAC knockdown and PINK1 overexpression(Scale bar  =  20 µm); e) Co‐localization analysis of MitoTracker Green and LysoTracker Red, staining of nuclei using Hoechst 33 342 (blue) after PINK1 knockdown and WAC overexpression(Scale bar  =  20 µm); f) ARS staining and ALP staining reveal levels of MSC osteogenic differentiation following WAC knockdown and PINK1 overexpression. Quantification of the data is presented in the right panel; g) ARS staining and ALP staining were performed after PINK1 knockdown and WAC overexpression. Quantification of the data is shown in the right panel. All data are presented as the means ± SD, *n* = 9 per group. Statistical differences were determined using Student's *t*‐test or ANOVA. ns not statistically significant, ^**^ *p* < 0.01 and ^***^ *p* < 0.001.

To elucidate the mechanism through which WAC regulates mitophagy, we conducted Co‐IP and LC‐MS/MS analyses to identify downstream proteins interacting with WAC. Mass spectrometry results indicated that PINK1, a crucial protein in mitochondrial autophagy, forms a binding association with WAC (Supporting Information). Subsequently, we validated the interaction between endogenous WAC and PINK1 in MSCs through Co‐IP (**Figure** **4**a). Furthermore, our previous study revealed a significant reduction in PINK1 level induced by SiWAC, suggesting that WAC may regulate mitophagy levels and osteogenic differentiation of MSCs through PINK1.

We further tested the role of PINK1 in WAC‐mediated mitophagy. Examination of mitophagy levels indicated that overexpression of PINK1 reversed the inhibition of mitophagy induced by WAC knockdown (Figure 4b). Similarly, knockdown of PINK1 inhibited mitophagy, but this inhibition was not significantly reversed by WAC overexpression (Figure 4c). Co‐localization analysis of MTG and LTR further confirmed these findings, showing that the co‐localization of MTG and LTR was reduced after knockdown of WAC, whereas it was increased after overexpression of PINK1 (Figure 4d). After knockdown of PINK1, co‐localization was decreased, whereas after overexpression of WAC, co‐localization of MTG and LTR did not change significantly (Figure 4e). Immunofluorescence showed the same change in the fluorescence intensity of LC3 (Figure S5a,b, Supporting Information). These results confirm that WAC positively regulates mitophagy during MSC osteogenesis through PINK1.

Subsequently, we further investigated whether WAC influences the osteogenic differentiation of MSCs through PINK1. The results demonstrated that the overexpression of PINK1 could counteract the negative regulatory impact of WAC knockdown on the strength of ARS staining and ALP activity in MSCs. Notably, the strength was markedly diminished upon PINK1 knockdown. Nevertheless, the overexpression of WAC failed to reverse the inhibitory effect of PINK1 knockdown (Figure 4f,g). We also examined the levels of PINK1 in MSCs from OP patients and showed that PINK1 protein levels were significantly decreased (Figure S6, Supporting Information). These results indicate that PINK1 serves as a crucial downstream mediator in MSC osteogenesis regulated by WAC.

### 2.5 WAC Inhibits Proteasomal Degradation of PINK1 by Regulating the Level of Ubiquitination at K137

**Figure 5:**
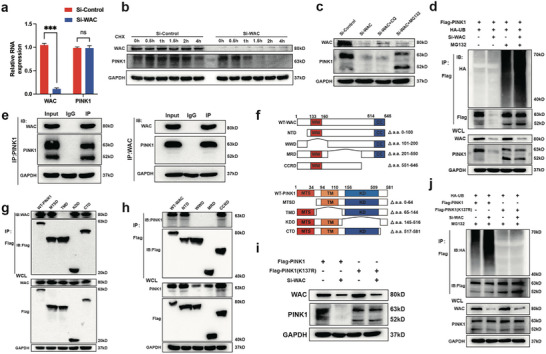
WAC inhibits proteasomal degradation of PINK1 by regulating the level of ubiquitination at K137. a) qRT‐PCR was employed to detect the relative mRNA expression of WAC and PINK1; b) WAC knockdown in MSCs using SiRNA, followed by protein lysate preparation at specified time points after treatment with 10 µm cycloheximide. PINK1 protein levels were assessed by Western blotting; c) Knockdown of WAC in MSCs using SiRNA, with subsequent treatment with lysosomal inhibitor (CQ, 10 mm) and proteasome inhibitor (MG132, 10 µm) for 12 h. PINK1 protein levels were detected by Western blotting; d) Overexpression of PINK1 and ubiquitin, followed by WAC knockdown and treatment with MG132. Immunoprecipitation using anti‐Flag was performed, and the level of PINK1 ubiquitination was detected by Western blotting; e) After treatment with MG132, immunoprecipitation assays using WAC, PINK1, or IgG antibodies on MSCs cell lysates. Protein levels of WAC and PINK1 were then detected by Western blotting; f) Diagram showing the missing structural domains and sequence of WAC and PINK1 deletion mutants; g) Transfection of WT‐PINK1 and PINK1 deletion mutants into 293T cells, followed by immunoprecipitation using anti‐Flag. WAC was detected in the immunoprecipitated complexes by Western blotting; h) Transfection of WT‐WAC and WAC deletion mutants into 293T cells, with subsequent immunoprecipitation using anti‐Flag. PINK1 was detected in the immunoprecipitated complexes by Western blotting; i) After knockdown of WAC, PINK1 (K137R) mutants were transfected into MSCs. PINK1 protein levels were detected by Western blotting; j) WAC was knocked down and PINK1 (K137R) mutants were transfected into MSCs, followed by treatment with MG132 for 12 h. The level of PINK1 ubiquitination was detected by Western blotting. All data are presented as the means ± SD, *n* = 9 in (a). Statistical differences were determined using Student's *t*‐test. ns not statistically significant and ^***^ *p* < 0.001.

We further investigated the mechanism of WAC regulating the level of PINK1. The mRNA level of PINK1 was not affected by WAC knockdown (**Figure** **5**a) while our above experiments indicated that WAC was integrated with PINK1 and capable of regulating the protein level of PINK1 (Figures 3a and 4a). This suggests that WAC is likely to regulate the change in PINK1 level exclusively at the protein level.

Protein stability is the crucial factor affecting the protein level thus we hypothesized that WAC might affect the degradation rate of PINK1. We employ cycloheximide (CHX) to inhibit protein synthesis, and further investigate the degradation of PINK1 protein after WAC knockdown. The results showed a faster rate of PINK1 degradation after WAC knockdown (Figure 5b). Considering that proteins are mainly degraded through the lysosomal or ubiquitin‐proteasome pathway, we blocked these pathways using chloroquine (CQ) and MG132, respectively. The PINK1 protein level decreased significantly after WAC knockdown compared to the control, with no significant change observed with CQ treatment. However, treatment with MG132 led to a significant increase in PINK1 protein level, suggesting that WAC regulates PINK1 level by influencing the proteasomal degradation pathway (Figure 5c). We co‐expressed HA‐Ubiquitin and Flag‐PINK1 in MSCs and observed that knockdown of WAC further increased the level of ubiquitination of PINK1 (Figure 5d). Moreover, we observed that knockdown of WAC enhances the binding of PINK1 to E3 ubiquitin ligase, indicating that WAC plays a protective role in preventing PINK1 ubiquitination (Figure S7, Supporting Information).

The ubiquitination‐proteasome pathway is one of the important protein degradation pathways in cells. In this pathway, proteins are ubiquitinated by binding to small ubiquitin proteins and are then sent to the proteasome for degradation. Previous studies have reported that PINK1 can be degraded after ubiquitination.^[^ ^31^ ^]^ The full‐length PINK1 undergoes mitochondrial transport mediated by MTS, and cleavage by presenilin‐associated rhomboid‐like protein (PARL) within the mitochondria to the 52 kD mature form.^[^ ^32^ ^]^ Subsequently, 52 kD PINK1 was degraded via the ubiquitination‐proteasome pathway.^[^ ^33^ ^]^ Similarly, after knocking down WAC, we observed that inhibition of the proteasome pathway resulted in a notable increase in the level of the 63 kD PINK1 protein and that the hidden 52 kD mature form of PINK1 could also be observed (Figure 5c).

To further explore the mechanisms, we subsequently investigated the interaction between WAC and various forms of PINK1. After treatment with MG132 and conducting Co‐IP, the findings revealed that WAC selectively interacts with the 63 kD form of PINK1, while showing no affinity for the 52 kD form (Figure 5e). To pinpoint the structural regions responsible for the interaction between WAC and PINK1, we designed various mutant plasmids based on their structural domains (Figure 5f). Co‐IP experiments with these mutants revealed that the WW domain and subsequent structural domains of WAC mediated its binding to the transmembrane (TM) domain of PINK1. Additionally, PINK1 protein levels were reduced in WAC deletions of the WW domain and intermediate regions (Figure 5g,h). We subsequently identified a key lysine residue K137 in the TM region of PINK1, which was reported by Liu et al. to be essential for ubiquitination degradation of the mature 52 kD form. We further constructed PINK1(K137R) mutation and found that the effects of WAC knockdown on the ubiquitination modification and protein level of PINK1 were abolished with K137R mutation (Figure 5i,j). In conclusion, these findings suggest that WAC inhibit proteasomal degradation of PINK1 by binding to the TM region of PINK1 and inhibiting K137 ubiquitination, thereby enhancing the protein level of PINK1.

### 2.6 WAC and PINK1 Help Improve Low Bone Mass in SAMP6 Mice and Promote Bone Defect Repair in Prx1‐Cre; WAC^fl/fl^ Mice

In order to unravel the regulatory role of WAC in osteogenic differentiation in vivo, we employed CRISPR‐Cas9 technology to generate WAC^fl/fl^ mice. Crossbreeding WAC^fl/fl^ mice with Prx1‐cre mice yielded Prx1‐Cre; WAC^fl/fl^ mice with WAC conditional knockout (CKO) specifically in MSCs (Figure S8a,b, Supporting Information). WAC expression assays in different tissues validated the specific knockdown of WAC in the skeletal system (Figure S8c, Supporting Information).

**Figure 6:**
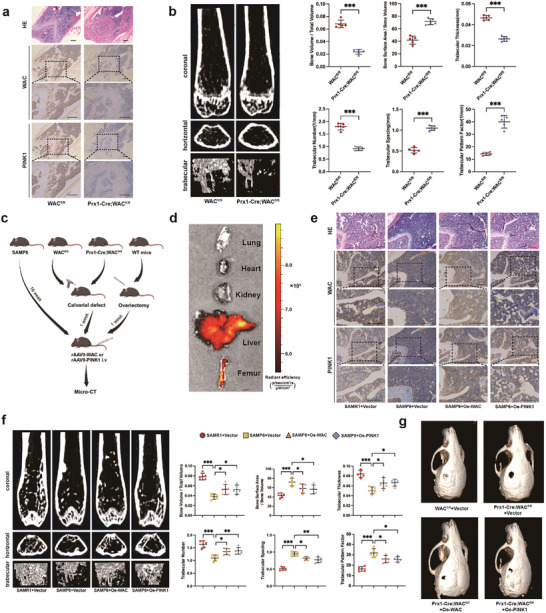
WAC and PINK1 help improve low bone mass in SAMP6 mice and promote bone defect repair in Prx1‐Cre; WAC^fl/fl^ mice. a) H&E staining and immunohistochemical staining for WAC and PINK1 in the femurs of WAC^fl/fl^ and Prx1‐Cre; WAC^fl/fl^ mice(Scale bar  =  100 µm); b) Micro‐CT analysis was conducted on WAC^fl/fl^ and Prx1‐Cre; WAC^fl/fl^ mice, capturing coronal and horizontal images of femurs. Three‐dimensional reconstruction of trabecular bones was performed, and bone morphometric analysis included parameters such as Bone Volume / Total Volume, Bone Surface Area / Bone Volume, Trabecular Thickness, Trabecular Number, Trabecular Spacing, and Trabecular Pattern Factor; c) Workflow of rAAV9‐WAC and rAAV9‐PINK1 injection to treat the SAMP6 mice, calvarial defect mice and OVX mice; d) Fluorescence images of organs in OVX mice injected with rAAV9‐WAC; e) H&E staining and immunohistochemical staining for WAC and PINK1 in the femurs of SAMR1 mice, SAMP6 mice, and SAMP6 mice injected with rAAV9‐WAC or rAAV9‐PINK1(Scale bar  =  100 µm); f) Micro‐CT analysis of SAMP6 mice treated with rAAV9‐WAC or rAAV9‐PINK1 injections. Images of the femur, including coronal and horizontal intercepts, were taken, and 3D reconstruction of bone trabeculae was performed. Bone morphometric analysis is presented in the right panel; g) Micro‐CT analysis depicting calvarial defects in WAC^fl/fl^ mice, Prx1‐Cre; WAC^fl/fl^ mice treated with the rAAV9‐WAC or rAAV9‐PINK1 injection. All data are presented as the means ± SD, *n* = 5 per group. Statistical differences were determined using Student's *t*‐test or ANOVA. ^*^ *p* < 0.05, ^**^ *p* < 0.01, and ^***^ *p* < 0.001.

Immunohistochemistry validated a significant decrease in the expression of both WAC and PINK1 in the femurs of Prx1‐Cre; WAC^fl/fl^ mice (**Figure** **6**a). Micro‐CT results illustrated that Prx1‐Cre; WAC^fl/fl^ mice displayed fewer and thinner trabecular size compared to WAC^fl/fl^ mice. 3D reconstruction of bone trabeculae confirmed bone volume and trabecular damage by measuring Bone Volume/Total Volume, Bone Surface Area/Bone Volume, Trabecular Thickness, Trabecular Number, Trabecular Spacing, and Trabecular Pattern Factor in the femurs of Prx1‐Cre; WAC^fl/fl^ mice (Figure 6b).

In order to explore the potential of targeting WAC and PINK1 to mitigate the progression of osteoporosis and promote bone formation, we designed the bone‐targeting recombinant adeno‐associated virus 9 (rAAV9) for the specific overexpression of WAC and PINK1 in bone tissue. Subsequently, rAAV9‐WAC and rAAV9‐PINK1 were injected into the tail vein of SAMP6 mice and calvaria defect mice (Figure 6c). The precise targeting of rAAV9 to the femurs was validated through fluorescence imaging of various organs (Figure 6d). Immunohistochemistry indicated a decrease in the expression levels of WAC and PINK1 in the femurs of SAMP6 mice, whereas the expression levels of both WAC and PINK1 increased in the femurs of rAAV9‐WAC‐treated SAMP6 mice. Notably, only the PINK1 expression level significantly increased in the femurs of SAMP6 mice after rAAV9‐PINK1 injection, and there was no significant change in the WAC level (Figure 6e). Moreover, in SAMP6 mice, injection of rAAV9‐WAC and rAAV9‐PINK1 resulted in a significant increase in bone density and mass, as shown by Micro‐CT (Figure 6f). The calvaria defect bone repair capacity of Prx1‐Cre; WAC^fl/fl^ mice was attenuated compared to WAC^fl/fl^ mice, and this attenuation was improved after treatment with rAAV9‐WAC and rAAV9‐PINK1 (Figure 6g). We also constructed OVX model to validate the effect of targeting WAC and PINK1 for the treatment of menopausal osteoporosis. The results showed that low levels of WAC and PINK1 existed in the femur specimens of OVX mice, and bone loss could be alleviated by injecting rAAV9‐WAC and rAAV9‐PINK1 (Figure S9a,b, Supporting Information). Together, these data indicated that WAC deletion leads to impaired bone formation in vivo and WAC and PINK1 are effective targets for the treatment of osteoporosis and bone defects.

## 3 Discussion

In this study, we illuminate the downregulation of WAC expression in osteoporosis and its pivotal influence on the osteogenic differentiation of MSCs in vitro. Our mechanistic investigations reveal that WAC forms a complex with the downstream target protein PINK1, acting as a safeguard against ubiquitination‐mediated degradation. This intricate interaction preserves mitophagy levels in MSCs, thereby stabilizing mitochondrial function and promoting osteogenic differentiation. Prx1‐Cre; WAC^fl/fl^ mice exhibited diminished bone mass and impaired bone defect repair, emphasizing the in vivo relevance of WAC in maintaining skeletal integrity. Notably, bone‐targeted rAAV9‐mediated overexpression of WAC and PINK1 ameliorated bone loss in SAMP6 premature aging mice. Our findings position WAC as a critical factor in MSCs, linking its deficiency to osteoporosis pathogenesis and underscoring its pivotal role in promoting osteogenic differentiation. This study suggests a promising way for innovative diagnostic and therapeutic strategies in osteoporosis management.

The WAC protein exhibits distinctive structural features, characterized by a WW domain at the N‐terminus and a coiled‐coil region at the C‐terminus.^[^ ^14^ ^]^ Previous studies have reported that WAC acts as a regulator of H2B ubiquitination and autophagy and participates in several physiological and pathological processes. Notably, WAC deletion has been associated with DeSanto‐Shinawi syndrome, a rare autosomal dominant disorder that manifests as mental retardation and learning disabilities.^[^ ^34^ ^]^ Furthermore, studies had linked mutations and deletions in the WAC gene to conditions such as growth retardation, unique cranial developmental abnormalities, as well as neurological disorders like autism and attention deficit disorder.^[^ ^35^ ^]^ However, few studies have investigated the role of WAC on bone metabolism. Here, we initiated an investigation into the relationship between WAC and osteoporosis, particularly its impact on MSC osteogenesis. We revealed a WAC deficiency in osteoporosis and uncovered the role of WAC in promoting MSC osteogenesis via PINK1‐mediated mitophagy. In addition, we showed the value of WAC in improving osteoporosis and bone defects in vivo. Consistently, previous studies also indicated an important role of WAC in cell differentiation.^[^ ^36^, ^37^ ^]^ Li’ study demonstrated an essential role of WAC‐mediated uH2B in plasma cell differentiation,^[^ ^36^ ^]^ and Karpiuk and his colleagues revealed that WAC coordinated H2Bub1 during MSC differentiation.^[^ ^37^ ^]^ While differently, our study is the first to systematically investigate the expression and mechanism of WAC in MSC osteogenesis and evaluate its application value on osteoporosis and bone repair.

Mitochondrial autophagy involves the selective degradation of damaged mitochondria through a receptor‐mediated mechanism, ensuring a healthy supply of intact mitochondria for energy production.^[^ ^37^ ^]^ Disruptions in mitophagy can disturb mitochondrial homeostasis, impacting cellular energy metabolism and physiological functions. Mitochondria play a crucial role in osteogenic differentiation, with increased mitochondrial biogenesis, function, and ATP content observed during osteoblast differentiation. Studies have highlighted that abnormal mitophagy levels contribute to bone metabolism disorders.^[^ ^25^ ^]^ Here, our data also indicate an effect of mitophagy on MSC osteogenesis and first identified WAC as a key factor engaging in mitophagy regulation. Our data revealed that WAC promoted mitophagy and MSC osteogenesis via maintaining the level of PINK1, and its effect on osteogenesis was reversed by mitophagy regulator. Lee observed that PINK1 deficiency‐induced osteogenic differentiation disorder was accompanied by reduced parkin levels, which suggested that WAC may regulate mitochondrial autophagy through the PINK1‐parkin axis to mediate the osteogenic differentiation of MSCs. Beyond the classical PINK1‐parkin pathway, numerous non‐parkin‐dependent mitophagy pathways exist. For instance, ARIH1 and SIAH1‐mediated pathways, which also involve PINK1 regulation, play roles in E3 ligase activity. Villa's research demonstrated that ARIH1 regulates mitophagy in cancer cells in a PINK1‐dependent manner,^[^ ^38^ ^]^ while Zhou's study found that the PINK1‐SIAH1 axis‐mediated mitophagy influences the sensitivity of hepatocellular carcinoma to sofilanib.^[^ ^39^ ^]^ However, the role of these related pathways in MSCs osteogenic differentiation has not yet been reported. We speculate that WAC‐regulated degradation of PINK1 may also impact these pathways, but further investigation is required to determine the activation of these pathways and their effects on MSCs osteogenic differentiation. In addition, WAC has been identified as a potent positive regulator of autophagy via interacting with GM130^[^ ^13^ ^]^ and is required for starvation‐induced autophagy.^[^ ^16^ ^]^ These results jointly suggest that WAC plays an important role in the regulation of autophagy and our study first revealed a role of WAC on mitophagy. Whether WAC participates in other kinds of autophagy such as reticulophagy or ribophagy requires further investigation.

PINK1, a protein kinase crucial for maintaining normal mitochondrial function, plays a pivotal role in the classical mitochondrial autophagy pathway known as the PINK1‐parkin pathway. Previous studies have focused on the role of PINK1‐mediated mitophagy on bone loss.^[^ ^41^ ^]^ Jang's study showed that PINK1 prevented osteoclast mitophagy impairment and therefore restrained excessive osteoclast differentiation and periodontitis‐induced bone loss.^[^ ^40^ ^]^ Wang's study indicated that PINK1‐mediated mitophagy enhanced osteogenic differentiation of periodontal ligament stem cells.^[^ ^42^ ^]^ Yuan and his colleagues revealed that PINK1‐mediated mitophagy modulates the production of cathepsin K in osteocytes and contributes to extracellular matrix degradation during bone loss.^[^ ^43^ ^]^ In addition, PINK1 inhibited apoptosis and promoted osteogenic differentiation of osteoblastic MC3T3‐E1 cells and might help improve age‐related osteoporosis.^[^ ^29^, ^44^ ^]^ Here, different from the above cell lines, we focused on MSCs and first uncovered the elevated expression and promoting function of PINK1 during MSC osteogenesis and its treating potential on bone loss and bone repair in vivo. Previous studies and our data jointly indicate that PINK1 has a wide range of osteogenic effects in the organism by regulating the functions of a variety of cells and plays a crucial role in bone formation. Accordingly, we believe that PINK1 is a highly potential target for the treatment of bone metabolism‐related diseases such as osteoporosis and bone defect, and deserves further efforts to explore its application value in clinical treatment.

**Figure 7:**
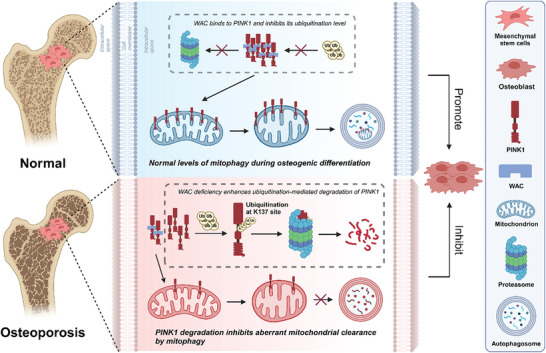
Schematic representation of the role of WAC in MSC osteogenesis. WAC binds to PINK1, shielding it from ubiquitination degradation, thereby maintaining normal levels of mitophagy and positively regulating the osteogenic differentiation of MSCs. The absence of WAC results in the ubiquitination degradation of PINK1 at the K137 locus and a reduction in mitophagy levels, leading to impaired MSC differentiation into osteoblasts.

WAC engages in diverse cellular functions through mediating protein‐protein interactions.^[^ ^45^ ^]^ WAC has been implicated in autophagy regulation by binding to the TTT and Pontin/Reptin complex to promote mTORC1 dimerization.^[^ ^46^ ^]^ Moreover, WAC interacts with Beclin1 and UBQLN4,^[^ ^16^ ^]^ both involved in the autophagy pathway. Here we first revealed that WAC interacted with PINK1 and protected it from ubiquitination‐mediated degradation. PINK1 plays a pivotal role in regulating mitochondrial autophagy and consists of three major structural domains: the mitochondrial targeting sequence (MTS), transmembrane domain (TM), and kinase domain (KM).^[^ ^47^ ^]^ Initially anchored to the mitochondrial membrane by MTS, PINK1 undergoes processing by mitochondrial processing peptidase (MPP) and subsequent cleavage between aa103 and 104 by PARL, resulting in its mature 52 kD form. In the context of damaged or depolarized mitochondria, dysfunctional PARL prevents cleavage, leading to PINK1 accumulation at the mitochondrial membrane and activation of the downstream mitochondrial autophagy pathway, ultimately promoting mitochondrial degradation.^[^ ^48^ ^]^ Conversely, in normal mitochondria, the 52 kD PINK1 is rapidly degraded to maintain mitochondrial integrity.^[^ ^31^, ^49^, ^50^ ^]^ Recent research has unveiled that the 52 kD form cleaved by PARL maintains low protein levels of PINK1 on mitochondria through ubiquitination at K137, facilitating degradation via the ubiquitin‐proteasome pathway.^[^ ^31^ ^]^ Nevertheless, Takatori's study fails to provide a good explanation as to why proteolytic removal by PARL between aa103 and 104 promotes the ubiquitination of PINK1 at the K137 locus. At the same time, no studies explore why full‐length PINK1 is not degraded by ubiquitination. Our study initially observed a basal level of PINK1 ubiquitination. Furthermore, PINK1 binding to E3 ubiquitination ligase was elevated after WAC knockdown, with a consequent increase in ubiquitination levels, suggesting that WAC depletion promotes ubiquitin‐mediated degradation of PINK1. Subsequent experiments revealed that WAC binds to the TM domain (aa 65–144) of PINK1 through its WW and MR domains, and WAC lacking these domains also facilitated PINK1 degradation. The K137 mutation in PINK1 not only eliminated ubiquitin‐mediated degradation under natural conditions but also abolished the increased ubiquitination promoted by WAC knockdown. This suggests that WAC knockdown may enhance ubiquitination specifically at the K137 site. Combining these results with the earlier observation of reduced full‐length PINK1 upon WAC knockdown and specific binding of WAC to full‐length PINK1, we propose that full‐length PINK1 interacts with WAC, inhibiting ubiquitination at the K137 site and preventing its degradation. Upon cleavage by PARL, exposing the K137 site and facilitating ubiquitination (**Figure** **7**). In conclusion, our study not only validates the significance of ubiquitination at the K137 site for PINK1 degradation but also reveals that WAC plays a crucial role in regulating PINK1 degradation by modulating K137 ubiquitination. This provides a novel explanation for the observed difference between the degradation rates of PINK1 in the cytosol and in mitochondria.

Our investigation extended to evaluate the potential of WAC and PINK1 as novel targets for the treatment of osteoporosis and bone repair. Initial observations in WAC‐CKO mice revealed disease manifestations akin to osteoporosis. Yeon‐Suk Yang achieved bone‐targeted expression of the target gene and anti‐osteoporosis by transplanting bone‐targeting peptide motif (AspSerSer)6 onto AAV9‐VP2 capsid protein.^[^ ^51^ ^]^ To further explore therapeutic interventions, we administered bone‐targeted rAAV9 vectors overexpressing WAC and PINK1 to SAMP6 and OVX mice. We observed similar specific delivery of rAAV9 in the femur. Notably, this intervention successfully reversed the low bone mass observed in SAMP6 and OVX mice which suggested that rAAV9 has important potential for use in bone‐specific regulation of gene expression in vivo and in the treatment of bone‐related diseases. Furthermore, we created a cranial bone defect model using WAC‐CKO mice and similarly administered bone‐targeted rAAV9 overexpressing WAC and PINK1. This intervention demonstrated a reversal of impaired bone defect repair in WAC‐CKO mice, emphasizing the potential of WAC and PINK1 as key targets in both osteoporosis treatment and bone restoration. In recent years, there has been significant research interest in small molecule agonists, which hold strong potential for clinical applications. Currently, there are few agonists targeting WAC or PINK1. Looking ahead, further investigation into the therapeutic potential of rAAV or small molecule agonists targeting WAC and PINK1 is warranted for clinical translation.

In summary, our study elucidates the significant role of WAC in modulating PINK1 degradation and influencing the osteogenic differentiation of MSCs. Nevertheless, our investigation has certain limitations. Whether WAC promotes PRAL cleavage of PINK1 is unclear, nor is the process by which WAC separates from PINK1; we speculate that PRAL plays a key role in this process, but further investigation is needed. Additionally, the lack of 3D structural analysis of WAC binding to PINK1 poses a challenge, warranting further research to comprehensively address these issues. Future studies aim to delve deeper into these aspects to enhance our understanding of the intricate molecular mechanisms involved.

## 4 Experimental Section

### Ethics Approval

The present study underwent thorough review and obtained approval from the Ethics Committee of the Eighth Affiliated Hospital of Sun Yat‐sen University, Shenzhen, China (2021d183, 2021r208). Stringent adherence to relevant regulatory standards was ensured throughout all experimental procedures. Before their involvement, all participants were provided with comprehensive information regarding the potential risks associated with the study and provided written consent.

### Isolation and Culture of MSCs

Fifteen individuals, aged between the age of 20 and 39, volunteered for this study. MSCs were isolated and cultured following established protocols.^[^ ^52^ ^]^ In brief, MSCs were extracted via bone marrow aspiration and subsequently purified using density gradient centrifugation. The cells were then suspended in Dulbecco's modified Eagle's medium (DMEM, glucose 1000 mg L^−1^, GIBCO) and seeded into culture flasks, where they were maintained at 37 °C with 5% CO_2_. The culture medium was refreshed every 3 days, and when cell confluence reached 80%, the cells were passaged using trypsin and reseeded into new flasks for expansion up to passage 4 for subsequent experiments.

For the clinical aspect of the study, five osteoporotic and five nonosteoporotic patients requiring surgery were recruited. Osteoporotic patients were defined as postmenopausal women with a lumbar bone mineral density (BMD) *T*‐value ≤ −2.5, while nonosteoporotic patients were nonmenopausal women with a BMD ≥ −1. Bone tissue was obtained during surgery, and MSCs were extracted from the bone marrow and cultured using the previously described procedure.

### Osteogenic Differentiation Induction

To induce osteogenic differentiation, osteogenic induction medium was prepared by adding 10% FBS, 100 IU mL^−1^ penicillin, 100 IU mL^−1^ streptomycin, 0.1 µm dexamethasone, 10 mm β‐glycerol phosphate, and 50 µm vitamin C to DMEM. MSCs were plated in 12‐well plates at a density of 0.8  ×  10^5^ cells/well and allowed to attach for 24 h. Subsequently, the culture medium was replaced with osteogenic induction medium, with subsequent medium changes performed every 3 days.

### Alizarin Red S (ARS) Staining and Quantification

MSCs were fixed using 4% paraformaldehyde for 30 min, followed by staining with 1% ARS solution for 15 min at room temperature, washed three times adequately using phosphate buffer solution (PBS) to remove non‐specific staining, and finally, the staining was observed under a microscope, and images were captured.

For quantitative assessment of ARS staining, after staining was completed, the cells were immersed in 10% cetylpyridinium chloride monohydrate (Sigma‐Aldrich) solution for 1 h at room temperature, followed by measuring the spectrophotometric absorbance of the solution at 562 nm.

### ALP Staining and Activity Assay

Following fixation with the paraformaldehyde solution, the differentiated MSCs underwent staining using the BCIP/NBT alkaline phosphatase kit (Beyotime Biotechnology), followed by microscopic observation and photography.

For the assessment of ALP activity, cells were lysed using RIPA buffer (Beyotime Biotechnology), followed by centrifugation. The supernatants were then collected and incubated with the reaction buffer for 15 min at 37 °C. After the addition of the termination buffer, absorbance was measured at 405 nm. Moreover, the determination of total protein concentration was conducted using the Pierce bicinchoninic acid protein assay kit (Thermo Fisher Scientific).

### RNA Isolation and qRT‒PCR Analysis

For RNA extraction from MSCs, TRIzol (Invitrogen) was utilized, followed by cDNA synthesis using the PrimeScriptTM RT kit (Takara). Subsequently, qRT‐PCR was conducted on the LightCycler480 PCR system (Roche), with SYBR Premix Ex TaqTM (Takara) added. The relative expression levels of WAC and PINK1 were determined using the 2−∆∆Ct method. The Primers sequence used in this study are detailed in Table S1 (Supporting Information).

### Protein Extraction and Western Blotting

Cells were lysed using RIPA buffer containing 1% phosphatase and protease inhibitor, protein supernatant was collected by centrifugation. Protein concentration was measured using the BCA Protein Assay Kit and diluted to the same concentration. After adding the SDS‐PAGE loading buffer, the protein was separated by SDS polyacrylamide gel electrophoresis and transferred to polyvinylidene fluoride membranes. After incubating the membrane with skimmed milk for 1 h, the membrane was incubated in primary antibody overnight at 4 °C, and the next day was washed three times with Tris‐buffered saline‐Tween (TBST) before being incubated in the corresponding secondary antibody for 1 h at room temperature. After three washes, chemiluminescent HRP substrate was applied to detect the target protein bands, and densitometry analysis was performed using ImageJ software. The primary antibodies used were as follows: anti‐WAC (Merck Millipore, Cat. No. ABE472); anti‐Runx2 (Cell Signaling Technology, Cat. No. 12556S); anti‐Osterix (Abcam, Cat. No. Ab229258); anti‐Ocn (Abcam, Cat. No. Ab133612); anti‐GAPDH (Cell Signaling Technology, Cat. No. 5174S); anti‐PINK1 (Cell Signaling Technology, Cat. No. 6946); anti‐P62 (Abcam, Cat. No. Ab109012); anti‐VDAC (Cell Signaling Technology, Cat. No. 4661); anti‐TIMM23 (Cell Signaling Technology, Cat. No. 34 822); anti‐LC3B (Abcam, Cat. No. Ab192890); anti‐Flag (Cell Signaling Technology, Cat. No. 2368); anti‐HA (Cell Signaling Technology, Cat. No. 3724); anti‐Ubr1 (Abcam, Cat. No. Ab108215); anti‐Ubr2 (Abcam, Cat. No. Ab217069); anti‐Ubr4 (Abcam, Cat. No. Ab86738); anti‐MFN1 (Cell Signaling Technology, Cat. No. 14 739); anti‐DRP1 (Abcam, Cat. No. Ab19274); anti‐FIS1 (Abcam, Cat. No. Ab156865).

### Immunofluorescence Staining

MSCs were cultured on glass slides within well plates and then washed three times with PBS before fixation with paraformaldehyde. Subsequently, the cells were treated with Triton X‐100 for 15 min at room temperature and incubated with goat serum for 30 min. Primary antibodies against COL1 (Cell Signaling Technology, Cat. No. 66 948), LC3B (Abcam, Cat. No. Ab192890), or TOM20 (Abcam, Cat. No. Ab186735) were added and left to incubate overnight at 4 °C. After three PBS washes, anti‐rabbit IgG (Cell Signaling Technology, Cat. No. 44 133) or anti‐mouse IgG (Cell Signaling Technology, Cat. No. 4408) secondary antibodies were applied and incubated for an additional hour at room temperature. Nuclei were stained with 4′,6‐diamidino‐2‐phenylindole (DAPI), and samples were examined under a laser scanning confocal microscope, with image acquisition at 488 nm (green, LC3B), 555 nm (red, COL1 and TOM20), and 405 nm (blue, DAPI) wavelengths using an LSM 5 Exciter confocal imaging system (Carl Zeiss).

For confocal analysis, cells were co‐incubated with 200 nmol MitoTracker Green (Beyotime, Cat. C1048) and 75 nmol LysoTracker Red (Beyotime, Cat. C1046) for 30 min at room temperature and protected from light, followed by incubation with 1% Hoechst 33 342 (Beyotime, Cat. C1027) for 5 min at room temperature and protected from light. Finally, images were captured using a confocal imaging system (Nikon Eclipse Ni‐E).

### Cleavage Under Targets & Tagmentation (CUT&tag)

The CUT&Tag assay was executed utilizing the NovoNGS CUT&Tag 2.0 A high‐sensitivity kit (Novoprotein Scientific, Cat. N259‐YH01‐01A). Initially, cells were gathered and enriched with ConA magnetic beads, followed by dual washes with Dig‐wash Buffer. Subsequent to this, samples were subjected to overnight rotation at 4 °C after being incubated with primary uH2B antibodies. On the following day, the primary antibody was removed, and cells underwent three washes before a 1 h incubation with secondary antibodies. Following incubation, beads were washed three times, and cells were exposed to Protein A‐Tn5 transposome. After an additional three washes, cells underwent tagmentation buffer incubation for 1 h, followed by a termination step with 10% SDS for 10 min. Finally, DNA fragments were extracted using phenol and chloroform. Subsequently, the libraries were sequenced on the Illumina NovaSeq 6000 platform at Novogene (Beijing, China), and sequencing data were acquired.

### Coimmunoprecipitation (Co‐IP)

MSCs or 293T cells underwent collection and lysis using a modified RIPA lysate, followed by centrifugation. For nonspecific binding removal, a small quantity of magnetic beads was employed. Following this, magnetic beads were added, and the mixture was incubated at 4 °C for 4 h. Antibodies against PINK1 (Cell Signaling Technology, Cat. No. 6946), WAC (Merck Millipore, Cat. No. ABE472), Flag‐Tag (Cell Signaling Technology, Cat. No. 205 606), or their corresponding IgG controls (Cell Signaling Technology, Cat. No. 3452) were subsequently co‐incubated overnight. The subsequent day, beads were retrieved and washed. After resuspending the beads with RIPA lysate, they underwent a 3‐min boiling step, and Western blotting was performed in accordance with established procedures. To mitigate the impact of antibodies on Western blotting, a specialized secondary antibody (Abcam, ab131366) was utilized to minimize detection of the antibody's heavy and light chains.

### Mass Spectrometry

Following electrophoresis‐based protein separation, staining was performed using the Coomassie blue staining kit (Beyotime Biotechnology). Specific bands were then selected for analysis utilizing liquid chromatography with tandem mass spectrometry (LC‐MS/MS) carried out by Applied Protein Technology (APT) company.

### Transmission Electron Microscopy (TEM) Analysis

MSCs were rapidly harvested and fixed overnight using phosphate buffer containing 4% formaldehyde and 2.5% glutaraldehyde. After fixation, the samples were pre‐embedded in agarose and then fixed with phosphate buffer containing 1% OsO4 for 2 h at room temperature. Subsequently, the samples were dehydrated with increasing concentrations of ethanol at room temperature and incubated with acetone solution for 15 min. Finally, after a series of resin permeation, polymerization, ultra‐thin sectioning and staining, the ultrastructure of MSCs, such as mitochondria and autophagosomes, was observed and photographed using TEM.

### Flow Cytometry

After rapid cell collection, cells were incubated with 100 nmol TMRE (MCE, Cat. HY‐D0985A) and 1 µmol mitoSOX (Invitrogen, Cat. M36008), respectively, for 30 min at 37 °C. The cells were rinsed twice with PBS after the incubation was completed, and the samples were subsequently assayed for fluorescence intensity using a BD FACSCelesta Flow Cytometer.

### ATP Concentration Measurement

MSCs were collected and lysed using a modified RIPA lysate then centrifuged for separation. The supernatant was retained for protein concentration and ATP concentration. Twenty microliters of protein solution was added to the prepared ATP assay working solution (Beyotime, Cat. S0027), incubated for 2 min, and then the absorbance was measured by Thermo Fisher Varioskan LUX. The ATP concentration in the samples was calculated based on the standard curve, and results were finally expressed as µmol/µg of protein.

### Infection of SiRNA, Plasmid, Lentivirus, and rAAV9 Construction

All small interfering RNA (SiRNA) targeting WAC and PINK1, including negative controls, were procured from IGEbio (Guangzhou, China). MSCs were transfected with a mixture of Opti‐MEM serum medium, Lipofectamine RNAiMAX (Invitrogen), and SiRNA in well plates. After 12 h of transfection, the medium was replaced. Assessment of knockdown efficiency was conducted 48 h later (Figure S10, Supporting Information), and SiRNAs achieving knockdown efficiency of over 50% were selected for subsequent experiments. Notably, SiRNA‐mediated knockdown of the target genes was repeated on day 6 of osteogenic induction. The sequences of the SiRNA utilized in this study are detailed in Table S2 (Supporting Information).

All plasmids, including wild‐type and mutant variants of PINK1 and WAC, were procured from IGEbio. MSCs were transfected with Lipofectamine 3000 (Invitrogen), mixed with cDNA as per the manufacturer's instructions.

All lentiviruses, encompassing vector controls and lentiviruses overexpressing WAC and PINK1, were designed and synthesized by OBiO Technology (Shanghai). MSCs were transfected with DMEM medium containing lentivirus (MOI = 30) and 5 µg mL^−1^ of polybrene, followed by removal of the medium after 48 h for subsequent experiments.

The construction of bone‐targeted rAAV9‐WAC and rAAV9‐PINK1 followed the methodology outlined by Yang.^[^ ^50^ ^]^ In summary, the DNA sequence encoding the bone‐specific peptide motif DSS (Asp‐Ser‐Ser)6 was inserted into the capsid protein VP2 of AAV9 to generate the rAAV9 bone‐targeting vectors.

### Prx1‐cre; WAC^fl/fl^ CKO Mice

C57BL/6 Prx1‐cre mice were purchased from the Jackson Laboratory. C57BL/6 WAC^fl/fl^ transgenic mice were purchased from GemPharmatech to construct Prx1‐cre; WAC^fl/fl^ mice. The following PCR primers were used for genotyping WAC^fl/fl^: 5′ arm primers, forward, TCCATCTCTTCCAACAAGTTGAGT, and 5′ arm primers reverse, CACATCTGACTTCAAAGTTCTTGC. Only the 185‐bp DNA bands will be detected in wild‐type mice, and only the 248‐bp DNA bands will be detected in homozygous WAC^fl/fl^ mice. Both 185‐bp and 248‐bp PCR DNA bands will be detected in heterozygous mice (WAC^fl/−^). PCR primers for the Cre sequence were used to detect the Prx1‐cre transgene in Prx1‐Cre mice: Prx1‐cre forward, GCTCTGATGTTGGCAAAGGGGT, and Prx1‐cre reverse, AACATCTTCAGGTTCTGCGGG.

### SAMP6 Mice and SAMR1 Mice

A total of 5 SAMR1 mice and 15 SAMP6 mice at 16 weeks of age were used in the experiments. the SAMP6 mice were randomly divided into three groups, two of which were administered rAAV9‐WAC and rAAV9‐PINK1 by tail vein injection. The mice were sacrificed after 6 weeks and femur samples were collected for subsequent experiments. The relevant animal experiments have been approved by Shenzhen TOP Biotechnology company Laboratory Animal Management and Use Committee (Approval Number: TOPGM‐IACUC‐2024‐0157).

### Construction and Treatment of the Mouse Postmenopausal Osteoporosis Model (OVX)

Approval for OVX mice and calvarial defect model mice was diligently obtained from the Institutional Animal Care and Use Committee of Sun Yat‐Sen University (Approval Number: 2 023 000 460). Eight‐week‐old female mice were subjected to bilateral ovariectomy, while the control mice underwent sham surgery, involving the excision of adipose tissue exclusively. On the seventh day post‐ovariectomy, administration of rAAV9‐WAC and rAAV9‐PINK1 was performed via tail vein injection. After 8 weeks, the mice were sacrificed, and femoral specimens were harvested for subsequent experimentation.

### Mice Calvarial Defect Model

Using a motorized bone drill, cranial defects were induced in 8‐week‐old mice. Before the surgical procedure, the mice were anesthetized and the surgical site was sterilized. A precise skin incision was made, and the tissue was gently separated to reveal the skull. The creation of cranial defects was performed using an electric bone drill equipped with a 2.5 mm drill bit. Following a 1‐week interval, rAAV9‐WAC and rAAV9‐PINK1 were injected via the tail vein. After an 8‐week duration, the entire skull was harvested for Micro‐CT analysis.

### Micro‐CT Scanning

After isolation, femurs and skulls from mice were immersed in paraformaldehyde for 2 days to facilitate fixation. Following fixation, they underwent comprehensive scanning and analysis utilizing the Micro‐CT system (Siemens). Employing Micro‐CT analysis software, image slices were meticulously reconstructed to generate both 2D and 3D structures. Subsequently, a detailed analysis of trabecular morphology was conducted, assessing key parameters such as Bone Volume/Total Volume, Bone Surface Area/Bone Volume, Trabecular Thickness, Trabecular Number, Trabecular Spacing and Trabecular Pattern Factor.

### HE Staining and Immunohistochemistry

Femoral specimens were fixed in 4% paraformaldehyde for 2 days and subsequently decalcified in 10% EDTA for 2 weeks. After decalcification, specimens were dehydrated, embedded in paraffin, and sectioned. Sections were stained with hematoxylin and eosin (HE). Immunohistochemistry was performed with anti‐WAC antibody from Solarbio (Cat. No. K109282P) and anti‐PINK1 antibody from Proteintech (Cat. No. 23 274).

### Statistical Analysis

The data in our study were analyzed using GraphPad Prism (San Diego, CA, USA) and SPSS software (Chicago, IL, USA). All results are presented as means ± standard deviations (SD). Significant differences between two groups were analyzed using Student's *t*‐test. Differences among more than two groups were analyzed using one‐way or two‐way analysis of variance (ANOVA). Correlation analysis was conducted by calculating Pearson’ s correlation coefficient. Significance was established at *p* < 0.05.

## Conflict of Interest

The authors declare no conflict of interest.

## Supporting information



Supporting Information

Supporting Information

## Data Availability

The data that support the findings of this study are available from the corresponding author upon reasonable request.
