# Supplementary material for: WAC Facilitates Mitophagy‐mediated MSC Osteogenesis and New Bone Formation via Protecting PINK1 from Ubiquitination‐Dependent Degradation
Source: Adv Sci (Weinh). 2024 Nov 18;12(2):2404107. doi: 10.1002/advs.202404107 (PMC11727373; doi:10.1002/advs.202404107)
Supplement: Supplementary file 1 — Supporting Information [file ADVS-12-2404107-s002.docx]

**Supplemental Information**

**WAC Facilitates Mitophagy-mediated MSC Osteogenesis And New Bone Formation Via Protecting PINK1 From Ubiquitination-dependent Degradation**

*Shuai Fan, Jinteng Li, Guan Zheng, Ziyue Ma, Xiaoshuai Peng, Zhongyu Xie, Wenjie Liu, Wenhui Yu, Jiajie Lin, Zepeng Su, Peitao Xu, Peng Wang, Yanfeng Wu^*^, Huiyong Shen ^*^, Guiwen Ye^*^*

**﻿**

**The Supporting Information for this manuscript includes**

**Table S1.** Primers used for qRT-PCR.

**Table S2.** SiRNAs used for RNA interference.

**Figure S1.** The level of uH2B modification on key osteogenic factor DNA did not exhibit significant changes following the knockdown of WAC.

**Figure S2.** WAC regulates the osteogenic differentiation of MSCs through mitochondrial autophagy.

**Figure S3.** WAC regulates autophagic flow during osteogenic differentiation of MSCs.

**Figure** **S4.** WAC regulates mitochondrial dynamics and function.

**Figure** **S5.** WAC mediates autophagic flow via PINK1.

**Figure S6.** Reduced PINK1 protein levels in MSCs from osteoporosis patients.

**Figure** **S7.** WAC regulates the binding of PINK1 to E3 ubiquitin ligase. After knockdown of WAC and treatment with MG132.

**Figure** **S8.** Generation of conditional WAC knockout mice.

**Figure** **S9.** WAC and PINK1 help to improve osteoporosis and inhibit osteoclast differentiation in OVX mice.

**Figure S10.** Knockdown efficiency assay of SiWAC and SiPINK1.

**The other Supporting Information for this manuscript includes:**

**Data file.** Detection of proteins interacting with WAC in MSCs by mass spectrometry

**Table S1. Primers used for qRT-PCR**

| **Gene** | **Forward primer (5' - 3')** | **Reverse primer (5' - 3')** |
| --- | --- | --- |
| GAPDH | GGAGCGAGATCCCTCCAAAAT | GGCTGTTGTCATACTTCTCATGG |
| WAC | ACTGGTCTGAGCATATTAGCTCT | TGACTGCCATCTTGTTTGCTT |
| PINK1 | CCCAAGCAACTAGCCCCTC | GGCAGCACATCAGGGTAGTC |

**Table S2. SiRNAs used for RNA interference**

| **Target Gene** | **SiRNA name** | **SiRNA sequence (5' - 3')** |
| --- | --- | --- |
| Control | NC | UUCUCCGAACGUGUCACGUTT |
| WAC | Si-1 | CCAGUUACUCUCCACAAGATT |
| WAC | Si-2 | CCAGUGGAAUGGAAGACAATT |
| WAC | Si-3 | GUCGAACAGAAGUUUCACATT |
| PINK1 | Si-1 | GCCAGUACCUUUGUGUGAATT |
| PINK1 | Si-2 | GCUGGAGGAGUAUCUGAUATT |
| PINK1 | Si-3 | GAAGCCAUCUUGAACACAATT |

**Supplemental Figure Legends**

**
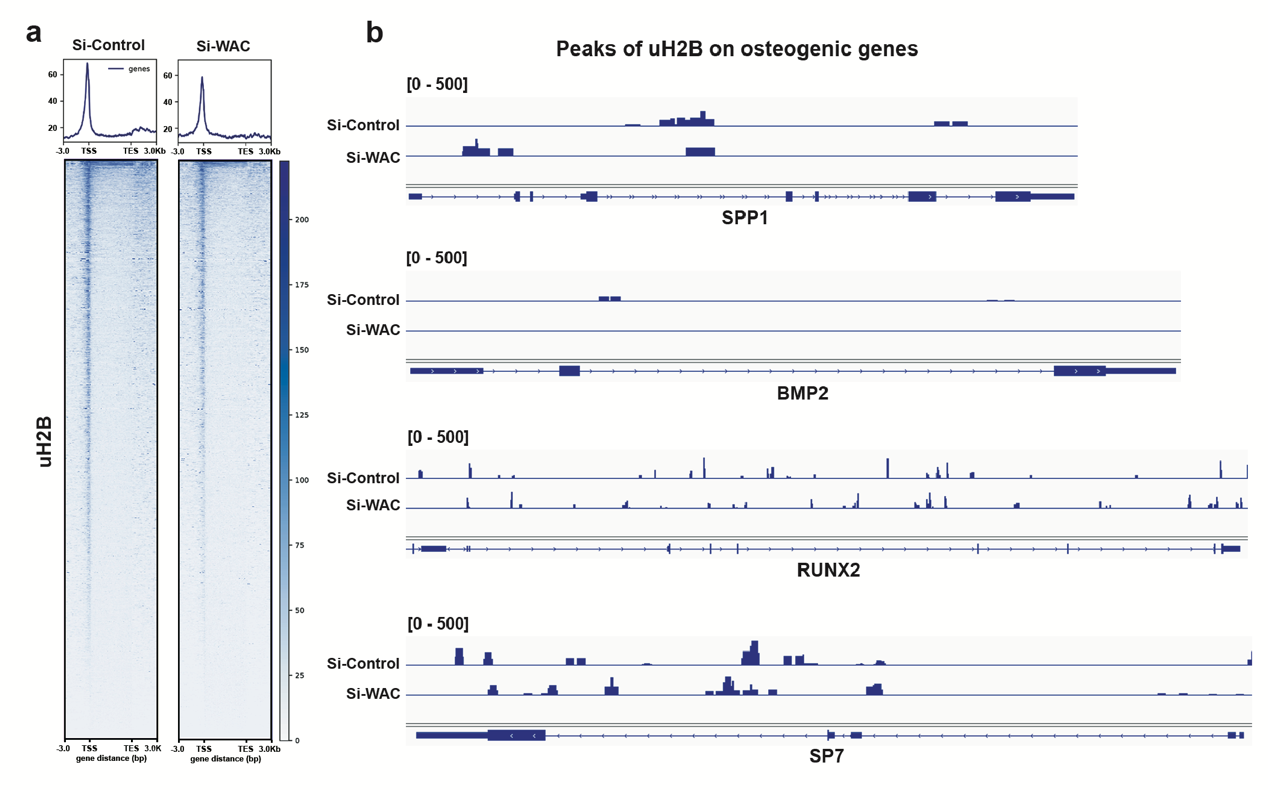
**

**Figure S1.** **The level of uH2B modification on key osteogenic factor DNA did not exhibit significant changes following the knockdown of WAC.** **a)** Heatmap of CUT&Tag curves of uH2B in MSC after knockdown of WAC; **b)** software analysis of the uH2B peaks of several key osteogenic factors（SPP1, BMP2, RUNX2, SP7）.


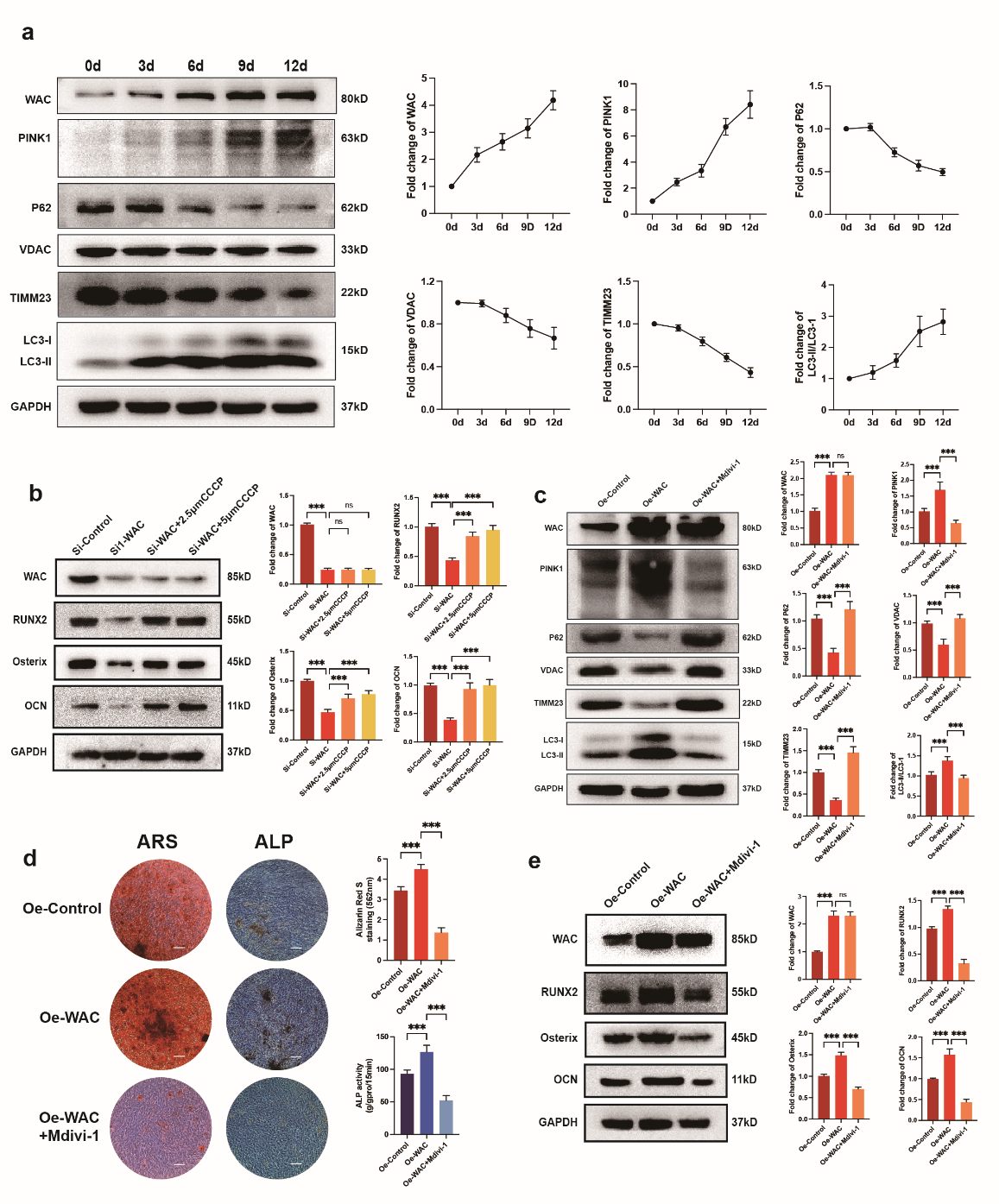


**Figure S2.** **WAC regulates the osteogenic differentiation of MSCs through mitochondrial autophagy. a)** Protein levels of WAC and mitophagy markers (PINK1, P62, VDAC, TIMM23, LC3B) during MSC osteogenic differentiation. Quantification is depicted in the right panel; **b)** Following knockdown of WAC and treatment with various CCCP, detection of protein levels of osteogenic markers; **c)** Protein levels of mitochondrial autophagy-related markers following overexpression of WAC and treatment with the mitophagy inhibitor Mdivi-1. Quantification of the data is shown in the right panel; **d)** ARS and ALP staining after overexpression of WAC and treatment with Mdivi-1; **e)** Detection of protein levels of osteogenic markers after after overexpression of WAC and treatment with Mdivi-1. Data quantification is illustrated in the right panel. All data are presented as the means ± SD, n = 9 per group. Statistical differences were determined using Student’s t test or ANOVA. ns not statistically significant and ***P < 0.001.


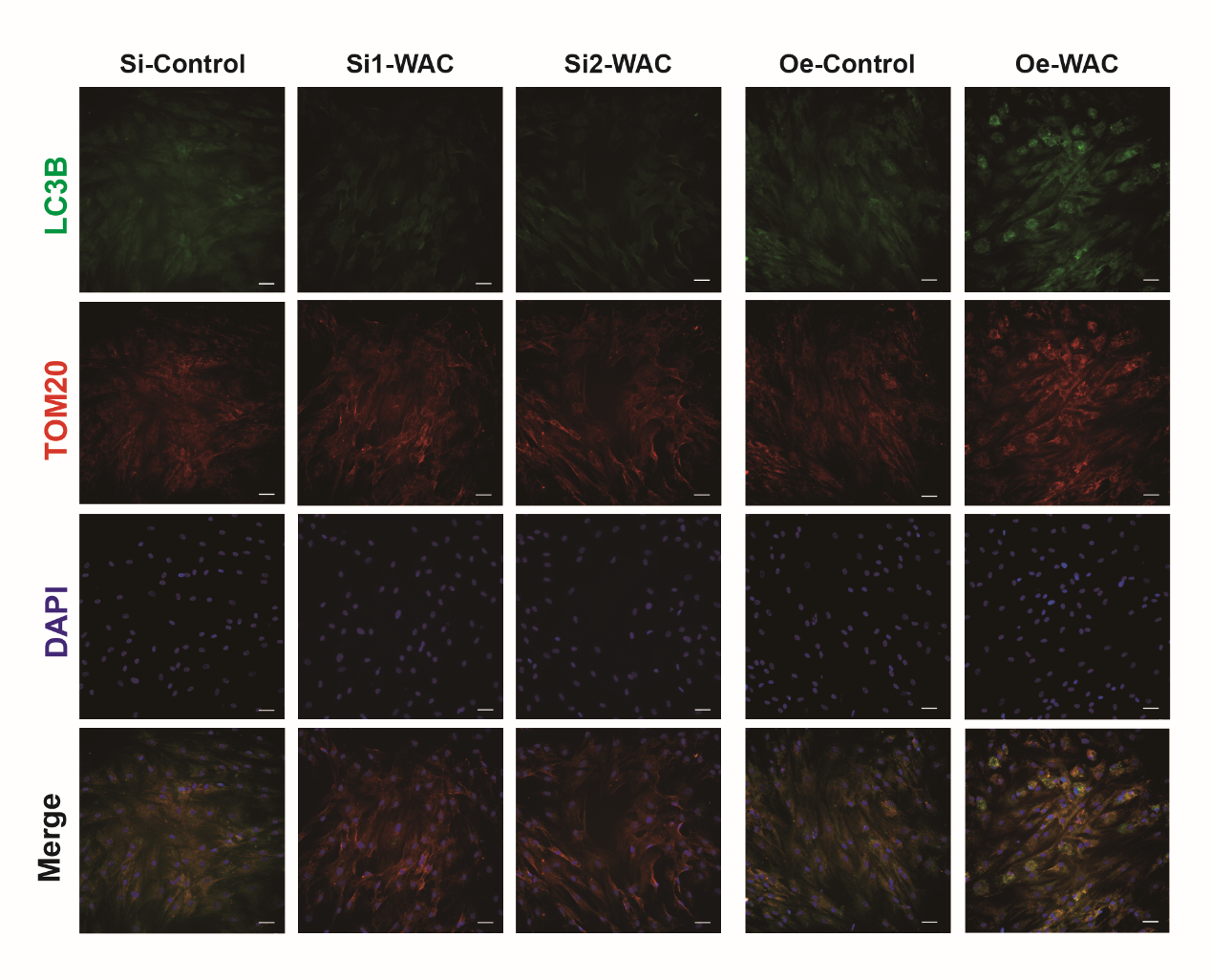


**Figure S3.** **WAC regulates autophagic flow during osteogenic differentiation of MSCs.** Immunofluorescence staining of LC3B (green) and TOM20 (red), with cell nuclei stained using DAPI (blue) (Scale bar = 50 μm);


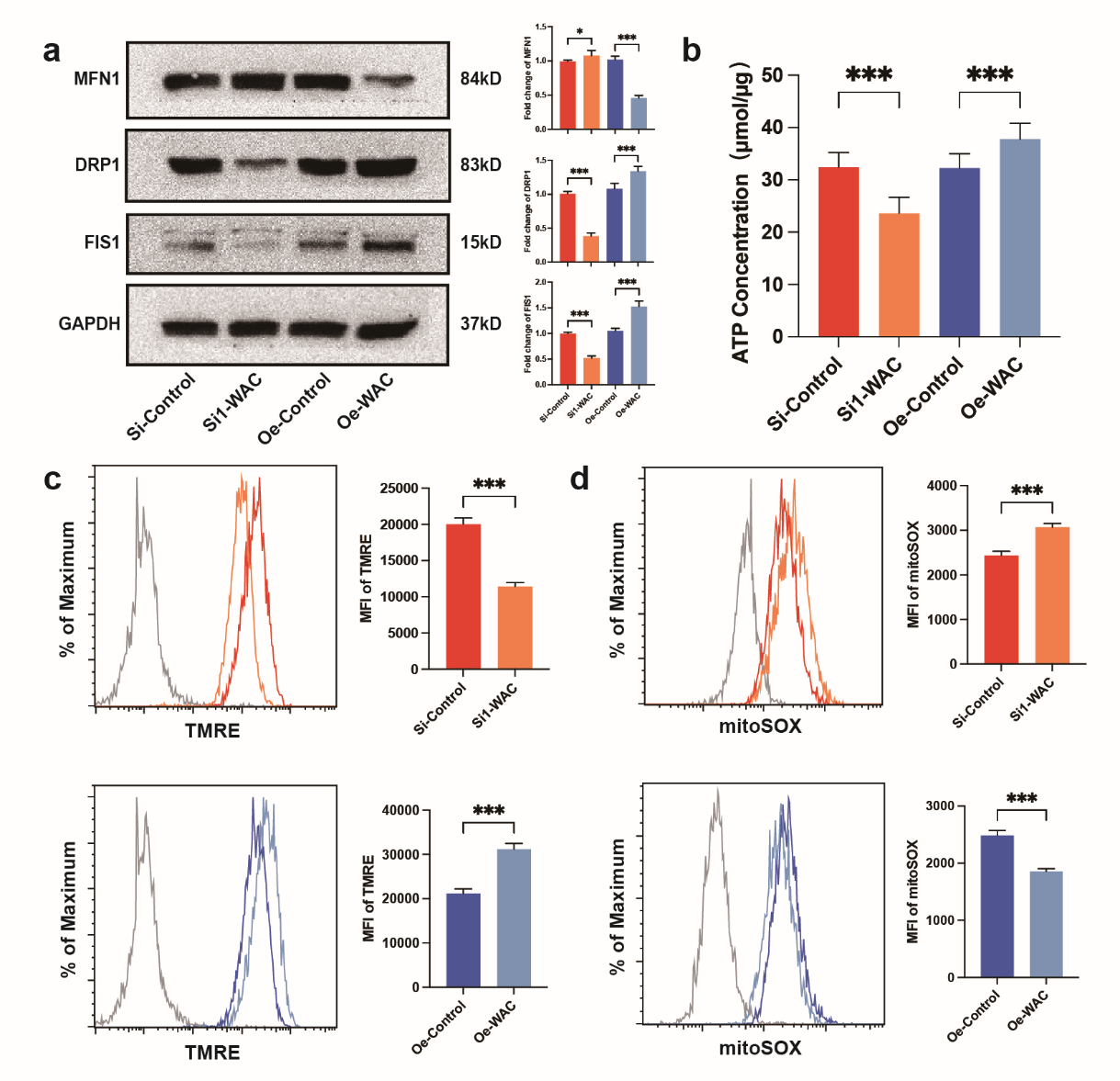


**Figure S4.** **WAC regulates mitochondrial dynamics and function. a)** Protein levels of the mitochondrial fusion-associated protein MFN1 and the mitochondrial fission-associated proteins DRP1 and FIS1 were assayed after WAC knockdown or overexpression. The right panel shows the quantitative results of the data; **b)** Detection of total intracellular ATP content after WAC knockdown or overexpression; **c)** After knockdown or overexpression of WAC, staining was performed using TMRE and fluorescence intensity was detected using flow cytometry; **d)** After knockdown or overexpression of WAC, staining was performed using mitoSOX and fluorescence intensity was detected using flow cytometry. All data are presented as the means ± SD, n = 9 per group. Statistical differences were determined using Student’s t test or ANOVA. ns not statistically significant and ***P < 0.001.


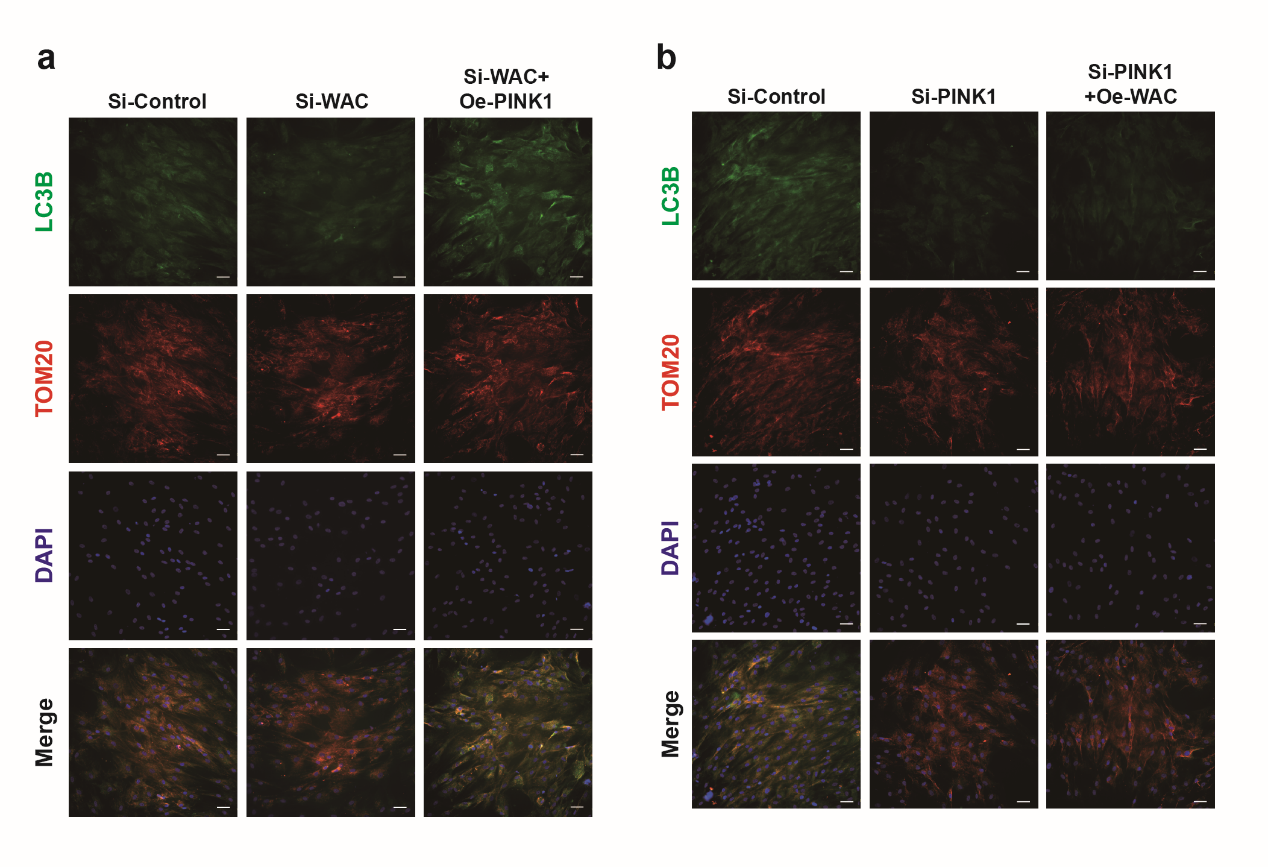


**Figure S5.** **WAC mediates autophagic flow via PINK1. a)** Immunofluorescence staining for LC3B (green) and TOM20 (red), with nuclei stained with DAPI (blue) after WAC knockdown and PINK1 overexpression(Scale bar = 50 μm); **b)** Immunofluorescence staining for LC3B (green) and TOM20 (red), with nuclei stained with DAPI (blue) after PINK1 knockdown and WAC overexpression(Scale bar = 50 μm).


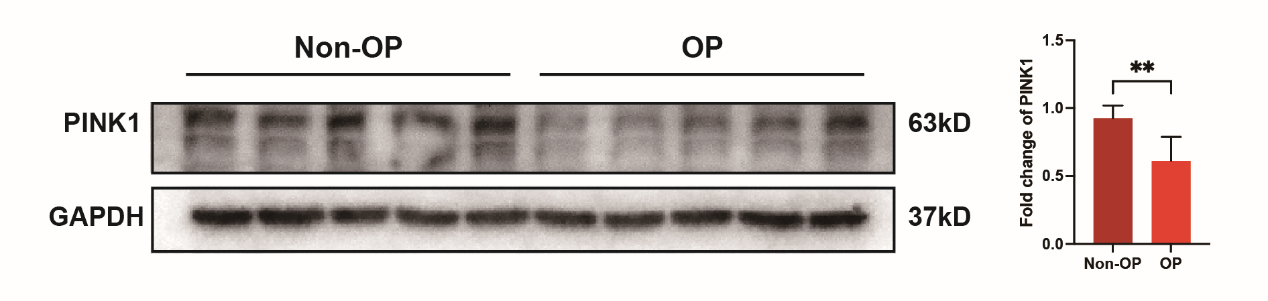
**Figure** **S6.** **Reduced PINK1 protein levels in MSCs from osteoporosis patients.** Western blotting to detect PINK1 protein levels in bone marrow MSCs from nonosteoporotic patients and patients with osteoporosis. All data are presented as the means ± SD, n = 9. Statistical differences were determined using Student’s t test. **P < 0.01.


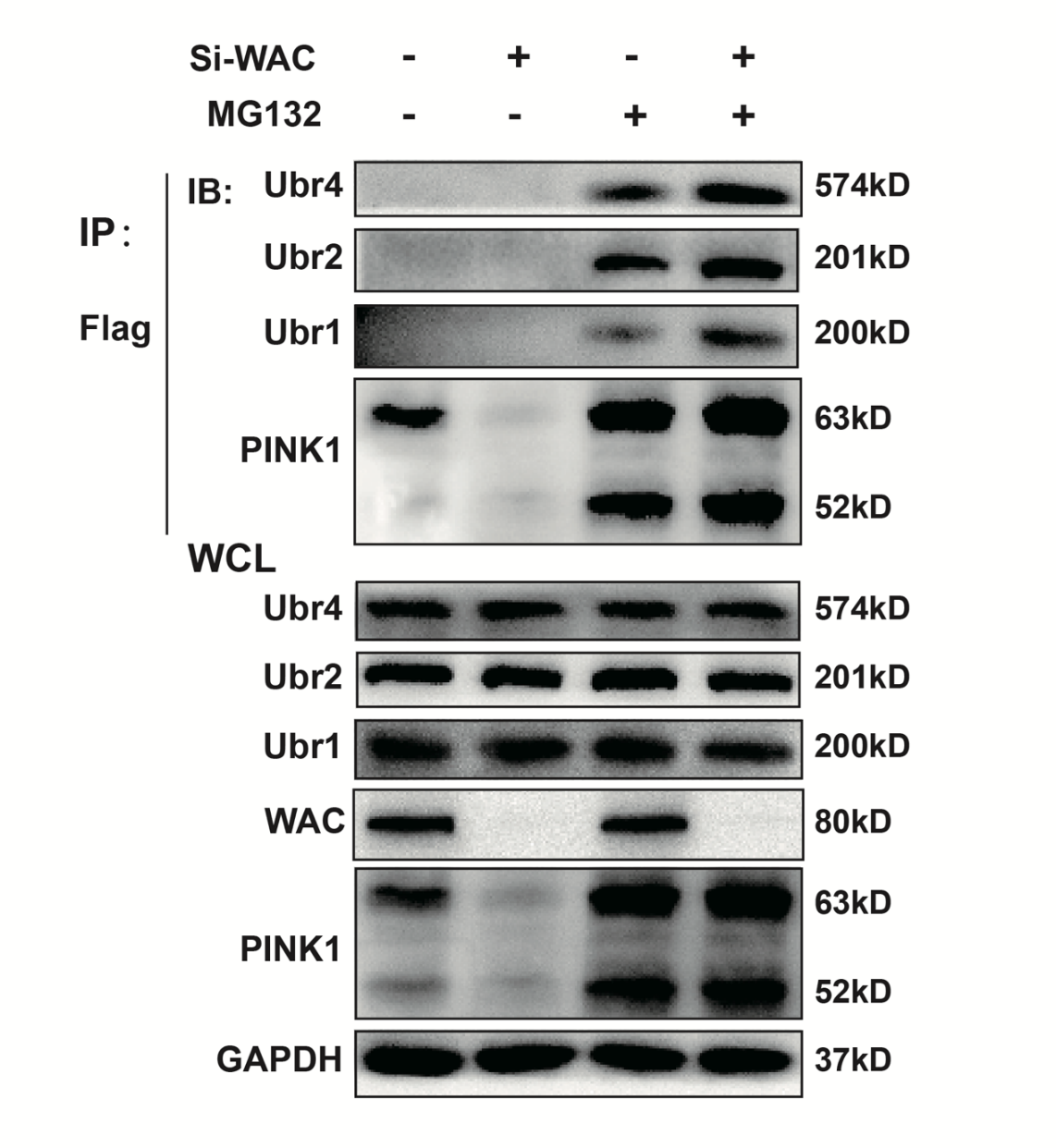


**Figure** **S7. WAC regulates the binding of PINK1 to E3 ubiquitin ligase. After knockdown of WAC and treatment with MG132.** Immunoprecipitation was performed using anti-PINK1 and Ubr1, Ubr2, and Ubr4 protein levels were detected by Western blotting.


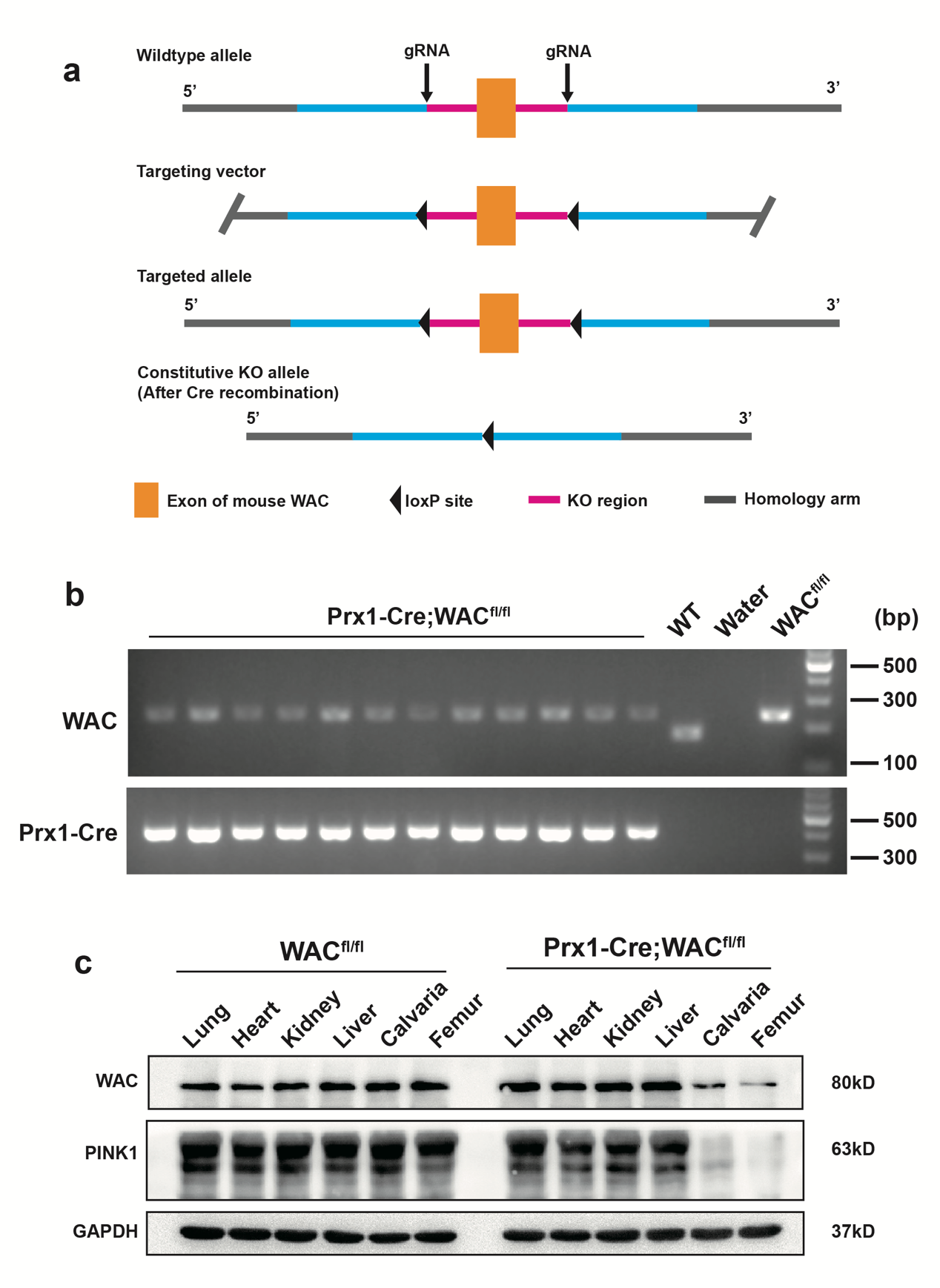


**Figure** **S8. Generation of conditional WAC knockout mice. a)** Schematic representation of the conditional WAC knockout strategy. Exon 1 was deleted after cre-mediated recombination, resulting in translation termination; **b)** Representative images of PCR genotyping; **c)** Western blotting showing the WAC and PINK1 protein levels in different organs of WAC^fl/fl^ and Prx1-Cre; WAC^fl/fl^ mice.


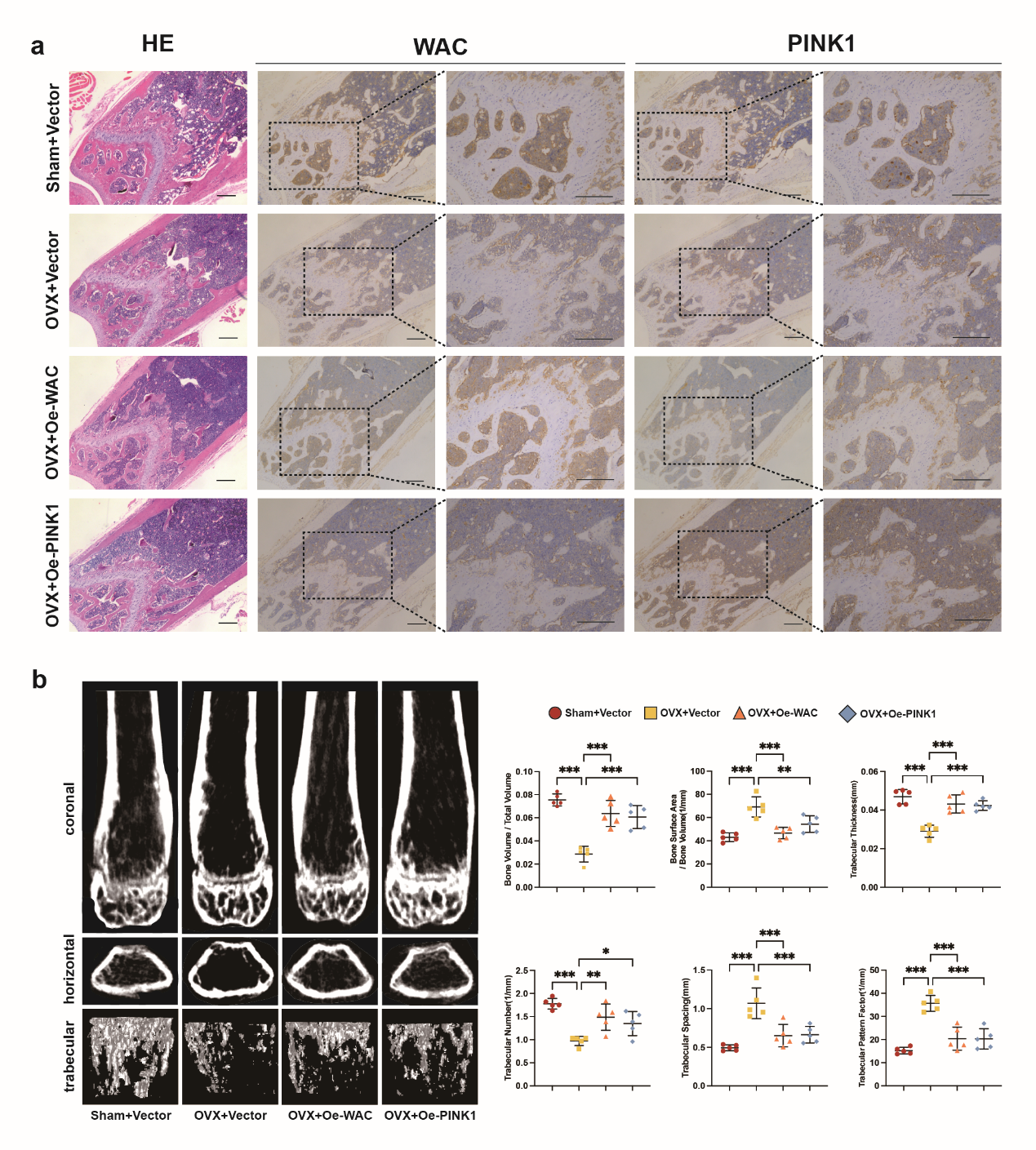


**Figure** **S9. WAC and PINK1 help to improve osteoporosis in OVX mice. a)** H&E staining and immunohistochemical staining for WAC and PINK1 in the femurs of the control group of mice, OVX mice, and OVX mice injected with rAAV9-WAC or rAAV9-PINK1(Scale bar = 100 μm); **b)** Micro-CT analysis of OVX mice treated with rAAV9-WAC or rAAV9-PINK1 injections. Images of the femur, including coronal and horizontal intercepts, were taken, and 3D reconstruction of bone trabeculae was performed. Bone morphometric analysis is presented in the right panel. All data are presented as the means ± SD, n = 5 per group. Statistical differences were determined using Student’s t test or ANOVA. *P < 0.05, **P < 0.01, and ***P < 0.001.


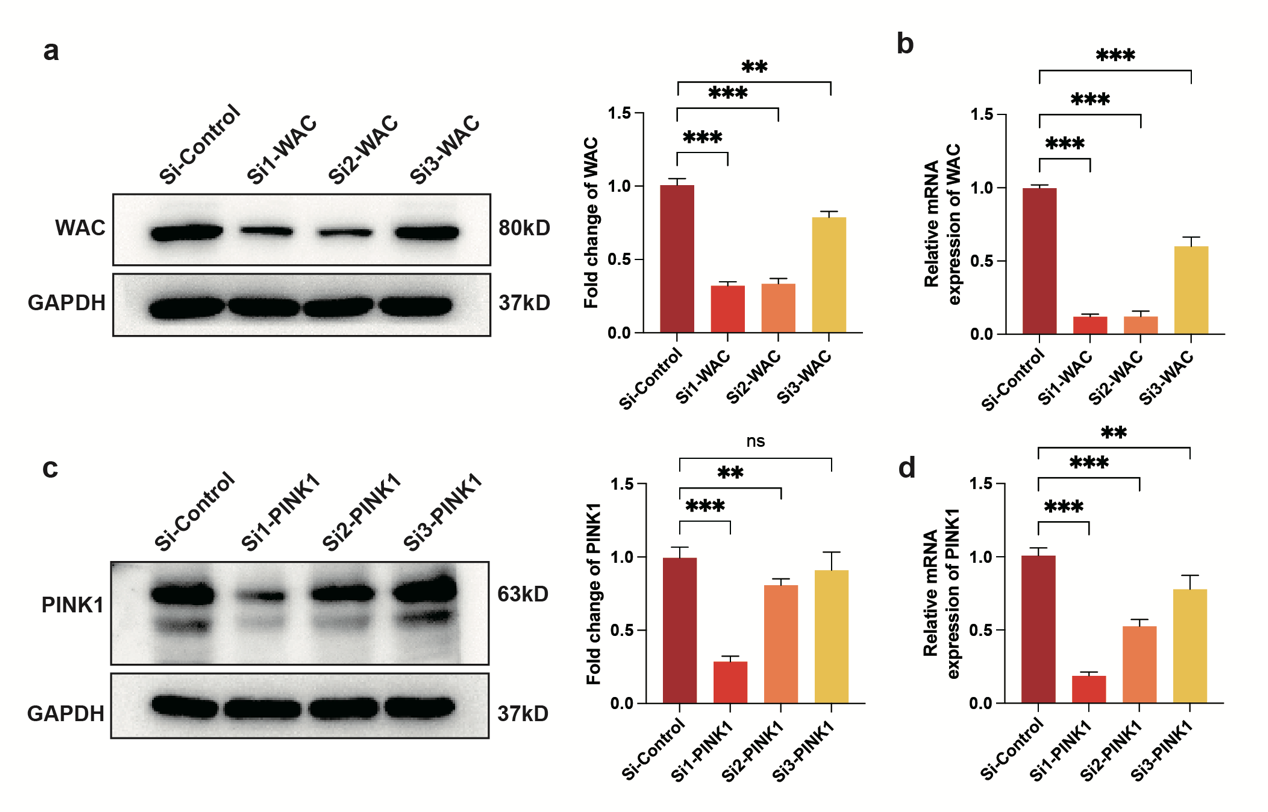


**Figure S10. Knockdown efficiency assay of SiWAC and SiPINK1. a)** The knockdown efficiency of SiWAC was assessed by Western blotting. **b)** SiWAC knockdown efficiency was measured using qRT-PCR. **c)** SiPINK1 knockdown efficiency was determined by Western blotting analysis. **d)** SiPINK1 knockdown efficiency was evaluated via qRT-PCR. All data are presented as the means ± SD, n = 9 per group. Statistical differences were determined using Student’s t test or ANOVA. ns not statistically significant, **P < 0.01, and ***P < 0.001.
